# Spatiotemporal deposition of cell wall polysaccharides in oat endosperm during grain development

**DOI:** 10.1093/plphys/kiad566

**Published:** 2023-10-20

**Authors:** Pavani P Nadiminti, Sarah M Wilson, Allison van de Meene, Alfie Hao, John Humphries, Julian Ratcliffe, Changyu Yi, Marta Peirats-Llobet, Mathew G Lewsey, James Whelan, Antony Bacic, Monika S Doblin

**Affiliations:** La Trobe Institute for Sustainable Agriculture & Food, Department of Animal, Plant and Soil Sciences, School of Agriculture, Biomedicine and Environment, La Trobe University, Bundoora, Victoria 3086, Australia; La Trobe Institute for Sustainable Agriculture & Food, Department of Animal, Plant and Soil Sciences, School of Agriculture, Biomedicine and Environment, La Trobe University, Bundoora, Victoria 3086, Australia; School of BioSciences, The University of Melbourne, Parkville, Victoria 3010, Australia; La Trobe Institute for Sustainable Agriculture & Food, Department of Animal, Plant and Soil Sciences, School of Agriculture, Biomedicine and Environment, La Trobe University, Bundoora, Victoria 3086, Australia; La Trobe Institute for Sustainable Agriculture & Food, Department of Animal, Plant and Soil Sciences, School of Agriculture, Biomedicine and Environment, La Trobe University, Bundoora, Victoria 3086, Australia; Latrobe University Bioimaging Platform, La Trobe University, Bundoora, Victoria 3086, Australia; La Trobe Institute for Sustainable Agriculture & Food, Department of Animal, Plant and Soil Sciences, School of Agriculture, Biomedicine and Environment, La Trobe University, Bundoora, Victoria 3086, Australia; La Trobe Institute for Sustainable Agriculture & Food, Department of Animal, Plant and Soil Sciences, School of Agriculture, Biomedicine and Environment, La Trobe University, Bundoora, Victoria 3086, Australia; La Trobe Institute for Sustainable Agriculture & Food, Department of Animal, Plant and Soil Sciences, School of Agriculture, Biomedicine and Environment, La Trobe University, Bundoora, Victoria 3086, Australia; La Trobe Institute for Sustainable Agriculture & Food, Department of Animal, Plant and Soil Sciences, School of Agriculture, Biomedicine and Environment, La Trobe University, Bundoora, Victoria 3086, Australia; La Trobe Institute for Sustainable Agriculture & Food, Department of Animal, Plant and Soil Sciences, School of Agriculture, Biomedicine and Environment, La Trobe University, Bundoora, Victoria 3086, Australia; La Trobe Institute for Sustainable Agriculture & Food, Department of Animal, Plant and Soil Sciences, School of Agriculture, Biomedicine and Environment, La Trobe University, Bundoora, Victoria 3086, Australia

## Abstract

Oat (*Avena sativa*) is a cereal crop whose grains are rich in (1,3;1,4)-β-D-glucan (mixed-linkage glucan or MLG), a soluble dietary fiber. In our study, we analyzed oat endosperm development in 2 Canadian varieties with differing MLG content and nutritional value. We confirmed that oat undergoes a nuclear type of endosperm development but with a shorter cellularization phase than barley (*Hordeum vulgare*). Callose and cellulose were the first polysaccharides to be detected in the early anticlinal cell walls at 11 days postemergence (DPE) of the panicle. Other polysaccharides such as heteromannan and homogalacturonan were deposited early in cellularization around 12 DPE after the first periclinal walls are laid down. In contrast to barley, heteroxylan deposition coincided with completion of cellularization and was detected from 14 DPE but was only detectable after demasking. Notably, MLG was the last polysaccharide to be laid down at 18 DPE within the differentiation phase, rather than during cellularization. In addition, differences in the spatiotemporal patterning of MLG were also observed between the 2 varieties. The lower MLG-containing cultivar AC Morgan (3.5% *w*/*w* groats) was marked by the presence of a discontinuous pattern of MLG labeling, while labeling in the same walls in CDC Morrison (5.6% *w*/*w* groats) was mostly even and continuous. RNA-sequencing analysis revealed higher transcript levels of multiple MLG biosynthetic *cellulose synthase-like F* (*CSLF*) and *CSLH* genes during grain development in CDC Morrison compared with AC Morgan that likely contributes to the increased abundance of MLG at maturity in CDC Morrison. CDC Morrison was also observed to have smaller endosperm cells with thicker walls than AC Morgan from cellularization onwards, suggesting the processes controlling cell size and shape are established early in development. This study has highlighted that the molecular processes influencing MLG content and deposition are more complex than previously imagined.

## Teaser

The spatiotemporal deposition pattern of mixed-linkage glucan (MLG), the most abundant cell wall polysaccharide, during oat endosperm development differs between high and low MLG varieties.

## Introduction

Oat (*Avena sativa*) is a cereal crop with a 5% annual growth in human consumption estimated over the next 5 years (Wilson et al. 2006; Cowman et al. 2021). This increase is closely associated with the health benefits gained by the consumption of oat grain whose cell walls, a major source of soluble dietary fiber, are particularly rich in the noncellulosic polysaccharides (1,3,1,4)-β-D-glucan (also known as β-glucan or mixed-linkage glucan [MLG]) and arabinoxylan (AX) (Collins et al 2010). Higher dietary (soluble and insoluble) fiber intake (∼25 to 30 g per day) is known to have multiple protective health benefits, for example, by reducing the risk of developing several chronic lifestyle-related diseases including obesity, type 2 diabetes, cardiovascular disease, stroke, colorectal cancer, and diverticulitis (Reynolds et al. 2019).

MLG is a homoglucan with strings of multiple (1,4)-β-D-glucosidic bonds that are randomly interspersed with single (1,3)-β-D-glucosidic linkages which create molecular “kinks’ within individual chains resulting in its enhanced solubility compared with cellulose (Fincher 2009). Dehulled oat grain (groats), contain between 2.3% and 8.5% *w*/*w* MLG (Welch and Lloyd 1989; Miller et al. 1995; Welch et al. 2000). In contrast, AX is a polymer of (1,4)-β-D-linked xylose units substituted to varying degrees primarily with arabinose residues and also with small amounts of glucuronic, acetyl, and phenolic acid (primarily ferulic acid) groups (Ebringerová et al. 2005). AXs constitute the second most abundant group of wall polysaccharides in oat grain walls, ranging between 2.2% and 4.1% *w*/*w* (Collins et al. 2010). MLG and AX together form the principal cell wall polysaccharides in cereals (family Poaceae).

Making up the bulk of the grain by weight, endosperm is the major nutritive component of cereal grain crops. Oat grain quality is known to be dependent on several factors such as the genetic pedigree of the cultivar, trait stability in response to epigenetic triggers, and effect of environmental factors not limited to temperature, drought, and soil fertility (Peterson et al. 1995; Stewart and McDougall 2014). Together, these factors pose a challenge to plant breeders in being able to generate oat varieties that are tailored to produce higher amounts of MLG and/or AX. To be able to breed cereal grains fit for purpose, it is important to understand the molecular mechanism of how nutrients and dietary fiber are assimilated and stored in the endosperm. Currently these processes are not well understood.

Endosperm development is well described in the major domesticated cereals, including wheat (*Triticum aestivum*) (Mares et al. 1975; Wegel et al. 2005), barley (*Hordeum vulgare*) (Wilson et al. 2006), rice (*Oryza sativa*) (Brown et al. 1996a, 1996b; Zhao et al. 2020), and maize (*Zea mays*) (Brown and Lemmon 2007; Sabelli and Larkins 2009). Briefly, endosperm development proceeds through a conserved pathway that starts with double fertilization in the megagametophyte (Olsen et al. 1999). A zygote is formed from the egg cell after its fertilization by 1 of the 2 sperm cells, while the other sperm cell fuses with the 2 polar nuclei, resulting in the formation of a triploid primary endosperm cell. The zygote forms an embryo that is typical of meristematic plants, and the primary endosperm enters a specialized pathway to develop into a nutritive tissue (Brown and Lemmon 2007). Of the 3 known developmental strategies for cellularization, ab initio cellular, helobial, and nuclear, ab initio is characterized by the formation of a cell wall after every endosperm nuclear division. In helobial endosperm development, nuclear division of the primary endosperm is followed by the formation of unequal micropylar and chalazal cells that develop independently (Floyd et al. 2000). The endosperm in grasses develops from a nuclear pattern where the multinucleate coenocytic syncytium formed after several nuclear divisions is devoid of cytokinesis and cell wall development (Mares et al. 1975; Wilson et al. 2006). A succession of anticlinal and periclinal wall formation events completes the process of endosperm cellularization (Palmer et al. 2015), and cellular differentiation leads to the formation of 4 different types of cells namely prismatic starchy endosperm, polygonal starchy endosperm, subaleurone, and aleurone (Mares et al. 1977; Sabelli and Larkins 2009).

However, despite oat being an important cereal crop, endosperm development and its associated molecular machinery, though assumed to be similar to other cereals within the Poaceae, has not been reported. While the nutritional profile of mature grain of a range of oat germplasm has been determined including dietary fiber components such as cellulose, MLG, and AX (Welch and Lloyd 1989; Miller et al. 1993; Westerlund et al. 1993; Miller et al. 1995; Peterson 1998; Kaur et al. 2019), critical information regarding the spatiotemporal accumulation of these dietary fiber components in oat endosperm cell walls is unexplored. In this study, we describe oat endosperm development as well as the timing and deposition pattern of cell wall components in 2 Canadian varieties with varying MLG abundance. The long-term goal of the work is to uncover the molecular events and machinery that lead to dietary fiber differences between the 2 varieties.

## Results

### Oat grain characterization

We investigated 2 commercially available Canadian spring varieties of oat, CDC Morrison, and AC Morgan. These lines were chosen for comparison as they differ in a number of agronomic traits and grain quality parameters. Data collected from 16 field sites in Canada and the United States over a 3-year period show that CDC Morrison grain contains more MLG than AC Morgan (Kibite and Menzies 2001) (Supplemental Table S1). However, AC Morgan has superior 1,000 kernel weight and generally displays higher yield than CDC Morrison.

Mature grain of AC Morgan and CDC Morrison were obtained from a 2020 oat variety trial conducted in Nipawin, Saskatchewan, Canada. Several biochemical assays were conducted to assess their macronutrient composition to verify the field data (see Supplemental Table S1). CDC Morrison grain was confirmed to contain a higher quantity of MLG (5.6% vs 3.5% AC Morgan), total dietary fiber (13.8% vs 12.6% AC Morgan), and protein (10.1% vs 8.6% AC Morgan), whereas AC Morgan grain showed higher starch levels (62.7% vs 57.6% CDC Morrison), contributing to its greater plumpness and kernel weight (Table 1).

**Table 1.** Nutrient composition (percentage by weight) of mature grain of Canadian oat spring varieties CDC Morrison and AC Morgan. Data are derived from this study using grain harvested in 2020 from a PepsiCo field site in Nipawin, Saskatchewan, Canada. Values are data averages ± Sd. See Supplemental Materials and Methods for details of each analysis

Grain component	CDC Morrison	AC Morgan
Starch content (%)	58.85 ± 0.82	61.75 ± 1.16
Total dietary fiber (%)	13.79 ± 0.85	12.62 ± 0.34
Protein (%)	10.05 ± 0.01	8.59 ± 0.04
MLG content (%)^a^	5.64 ± 0.09	3.51 ± 0.06
DP3/DP4 ratio^b^	1.82 ± 0.08	1.92 ± 0.06

^a^MLG assay (Megazyme) using groat flour.

^b^HPAEC-PAD analysis.

Monosaccharide linkage analysis of the “popped” endosperm (Gartaula et al. 2017) was used to deduce their cell wall polysaccharide composition (see Supplemental information). MLG was the most abundant wall polysaccharide with heteroxylan (HX) being the next most abundant (Table 2, Supplemental Tables S2 and S3). CDC Morrison endosperm walls were estimated to contain ∼2.5% to 3% more MLG than AC Morgan, similar to the difference determined by the standard Megazyme MLG assay (Table 1). Small amounts (<5%) of other wall polymers were detected including glucomannan, pectin, arabinogalactan-proteins (AGPs), cellulose, and callose; the latter 2 were verified by enzymatic digestion assays but did not vary substantially between the 2 oat varieties.

**Table 2.** Calculated polysaccharide composition of “popped” endosperm cell walls of mature CDC Morrison and AC Morgan grain. Calculations are based on the method outlined in Pettolino et al. (2012)

Polysaccharide		CDC Morrison	AC Morgan
		(mol %)	
(1,3;1,4)-β-Glucan^a^		71.37	68.14
(Glucurono)arabinoxylan		13.45	12.73
Cellulose		4.12	4.76
Xyloglucan		0.17	0.44
Glucomannan		1.99	2.17
Callose		1.75	2.28
AGPs	Type II AG	2.04	2.75
Pectin	Arabinan	0.61	0.79
	Type I AG	0.58	0.15
Other		3.93	5.79

^a^MLG assay (Megazyme): CDC Morrison, 77.01%; AC Morgan, 73.53%.

### Oat endosperm development

Grain development was followed in both CDC Morrison and AC Morgan (see Supplemental Fig. S1, A and B, for whole cross-sections of mature grain) from panicle emergence (day of heading, designated as 0 d) to maturity. We chose to monitor grain development from this stage rather than anthesis as it is easier and more accurate to identify since oat anthers aren’t always visible outside the floret at flowering. Both cultivars were observed to follow a near identical series of cellular events divided into 4 phases of development described below with very similar timing (Supplemental Fig. S2) despite the slightly earlier heading date of CDC Morrison (Supplemental Table S1). For this reason, we show microscopy data predominately from CDC Morrison grains except when differences between the 2 cultivars are highlighted.

### Syncytial

**Figure 1.**
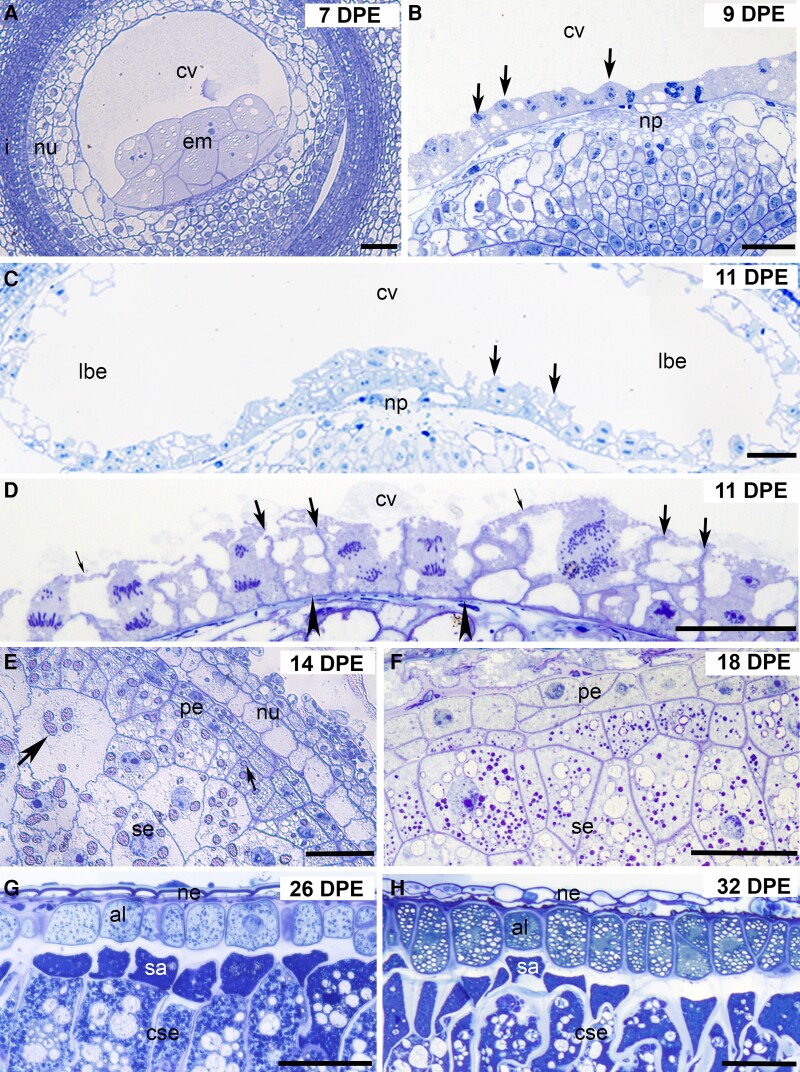
Light micrographs of oat grain sections stained with Toluidine Blue from 7 to 32 DPE. **A)** Syncytial stage of endosperm development at 7 DPE showing a large vacuolated central cell and multicellular embryo surrounded by the nucellus and integuments. **B)** A higher magnification image of the syncytial endosperm at 9 DPE showing nuclei (arrows) embedded within a thick layer of cytoplasm adjacent to the grain crease. **C)** At 11 DPE, cellularization has begun but only after the central cell has expanded to form the early distal lobes. The image shows the early walls (arrows) extending from the central cell wall. **D)** A high-magnification image of early cellularization showing actively dividing nuclei partitioned by anticlinal walls (large arrows) extending from the central cell wall (arrowhead) with an overlying layer of residual cytoplasm (small arrows). **E)** At 14 DPE, the endosperm is fully cellularized, but the walls are thin and wavy as large starch granules (large arrow) begin accumulating in the grain. Cells from the peripheral endosperm are smaller with fewer and smaller starch grains (small arrow). **F)** At 18 DPE, the peripheral endosperm consists of 2 to 3 small layers of rectangular cells with prominent nuclei and no starch granules. To the inside are larger cells packed with starch. **G)** At 26 DPE, the aleurone is 1 cell layer thick and consists of large rectangular cells devoid of starch each with a prominent nucleus. To the inside are the small, intensely stained subaleurone cells and large endosperm cells packed with starch. **H)** By 32 DPE, the aleurone cells have increased in size and the walls between neighboring aleurone cells have thickened. al, aleurone; cse, central starchy endosperm; cv, central vacuole; em, embryo; i, integuments; lbe, lobe; np, nucellar projection; nu, nucellus; se, starchy endosperm; pe, peripheral endosperm. Scale bars = 50 *μ*m.

Evidence of the central cell was first observed at around 7 days postemergence (DPE) when a small region of vacuole and a multicellular embryo was detected in transverse sections (Fig. 1A). Over the next 2 d, this vacuole expanded and at ∼9 DPE numerous nuclei were seen lining the perimeter of the central cell within a continuous, thin layer of cytoplasm (Fig. 1B, arrows). Before any walls are laid down, the oval-shaped central cell widens to form the early distal lobes that are connected by a narrower region of vacuole termed the central endosperm (Fig. 1C). The ventral side of the central endosperm lies adjacent to the maternal vasculature, and this region is referred to as the grain crease (Supplemental Fig. S1, A and B). The syncytial cytoplasm on the ventral side of the crease is much denser than in other regions of the developing syncytium, and the cells formed in this region will become specialized transfer cells tasked with providing nutrition to the growing grain. The outward appearance of the grain changed in the first few days after panicle emergence by first widening, coinciding with vacuole expansion, and then elongating before the endosperm moves into the next phase of development.

### Cellularization

At ∼11 DPE, the first-formed anticlinal walls are laid down and extend from the central cell wall to form partitions between nonsister nuclei termed alveoli: tube-like cell walls encasing individual nuclei with an open end facing the central vacuole (Fig. 1, C, arrows, and D, large arrows). The nuclei in turn divide almost synchronously, and periclinal walls are deposited between the sister nuclei, resulting in the first layer of “cellularized” endosperm and a new layer of alveoli with an overlying layer of residual syncytial cytoplasm (Fig. 1D, small arrows). This process occurs ∼5 to 6 times until the endosperm is fully cellularized. The progression from the syncytium to a fully cellularized endosperm in oats occurs quite rapidly because we rarely encountered grains part-way through cellularization despite sectioning hundreds of grains that were of the appropriate age and appearance for the cellularization phase. Nevertheless, we detected that the central endosperm, which is the narrowest region of the grain, is completely cellularized well ahead of the lobes and that the peripheral endosperm cells enter the differentiation phase before cellularization is complete at ∼13 DPE.

### Differentiation

We selected 3 timepoints, 14, 18, and 26 DPE, to follow the differentiation phase in oat endosperm. This phase is marked by the development of 5 specialized cells/tissues including the aleurone and transfer layer and the 3 types of starchy endosperm cells, polygonal, prismatic, and subaleurone. At 14 DPE, the early, thin cell walls of the “cellularized” endosperm very rapidly thicken and straighten as cell wall polysaccharides are deposited. Large starch granules can be seen in the cells located in the middle of the endosperm, while the smaller cells in the 3 to 4 layers positioned at the periphery accommodate fewer starch granules. These peripheral cells contain dense cytoplasm and numerous small vacuoles (Fig. 1E).

By 18 DPE, 2 distinct lobes have formed, connected by a narrower region of central endosperm. By this stage, the maternal layers including the integuments have mostly disintegrated but areas of crushed nucellar tissue still adhere to the outward-facing periclinal cell walls of the differentiating peripheral endosperm. The 2 to 3 cell layers in this region are small, ordered, and rectangular with visible cytoplasm but no starch granules. To the inside of the peripheral endosperm are large, irregularly shaped and undifferentiated starchy endosperm cells packed with amyloplasts (Fig. 1F).

At 26 DPE, the differentiation phase is complete. The aleurone is 1 cell layer thick, and the cells are rectangular in shape, often with prominent nuclei and filled with protein bodies or “aleurone grains” (Fig. 1G). The walls of the aleurone are thicker than elsewhere in the grain, particularly the inner periclinal walls that face toward the starchy endosperm. By 26 DPE, the starchy endosperm has differentiated into 3 cell types: subaleurone, prismatic, and polygonal. The subaleurone is the single layer of cells immediately adjacent to the aleurone, arising from a periclinal division of the outer endosperm cell layer late in the differentiation phase. These are the smallest cells of the starchy endosperm and are characterized by an irregular shape and thick cell walls disproportionately thicker on the side that face the aleurone layer (Fig. 1G). The prismatic starchy endosperm cells are evident by 26 DPE and are located in the central region of the grain adjoining the 2 lobes. These prismatic cells (Supplemental Fig. S1C) have a narrow, elongated shape in contrast to the polygonal-shaped cells that occupy the lobes (Supplemental Fig. S1D).

### Maturation

The final phase of endosperm development is defined by further starch (and protein) accumulation, programmed cell death, and desiccation. By the end of the maturation phase, the only tissues that remain living are the aleurone, transfer cell layer, and maternal vasculature. At 32 DPE, the walls of the aleurone cells have thickened further as have the walls of the subaleurone, most prominently those at the aleurone/subaleurone interface (Fig. 1H). Each rectangular peripheral aleurone cell has a prominent nucleus surrounded by numerous protein bodies that were intensely stained with Toluidine Blue. The subaleurone cells are irregular in shape and contain protein bodies and small starch granules. The prismatic cells in the central starch endosperm have lengthened and continue to accumulate starch as do the irregular polygonal cells located within the lobes (Supplemental Fig. S1, A to D).

### Comparison of AC Morgan and CDC Morrison grain structure

**Figure 2.**
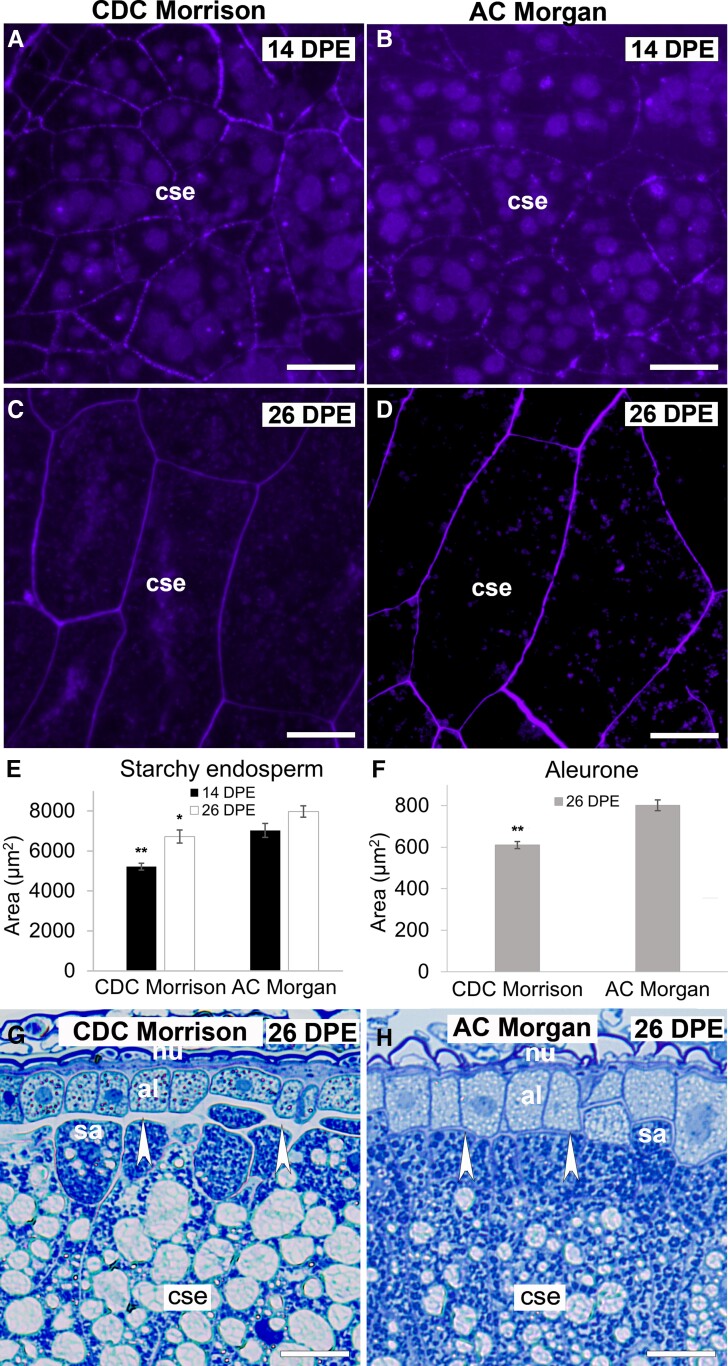
Comparison of endosperm and aleurone cell size in CDC Morrison and AC Morgan grains at 14 and 26 DPE. Cross-sections of starchy endosperm cells of AC Morgan grain at 14 DPE **A)** and 26 DPE **C)** stained with Calcofluor White. CDC Morrison cells of the same age shown in **B)** and **D)**, respectively. Average cell area ± Se of starchy endosperm (14 DPE, *n* = 60 per cultivar; 26 DPE, *n* = 90 per cultivar) **E**) and aleurone (26 DPE; *n* = 90 per cultivar) **F**) cells. Asterisks = *P* < 0.001 (Mann–Whitney *U* test). **G)** and **H)** The difference in thickness of the periclinal wall at the aleurone/subaleurone interface in CDC Morrison **G)** and AC Morgan **H)** grain at 26 DPE is indicated by arrowheads. al, aleurone; cse, central starchy endosperm; nu, nucellus; sa, subaleurone. Scale bar = 50 *μ*m (**A** to **D**), 20 *μ*m (**G** and **H**).

Previously, Miller and Fulcher (1994) showed that differences in MLG content were reflected in the size and wall thickness of endosperm cells. A high MLG-containing oat cultivar, Marion (6.4% *w*/*w*), had smaller cells with slightly thicker walls than the lower MLG cultivar, OA516-2 (4.0% *w*/*w*). To determine if a similar trend was observed in CDC Morrison and AC Morgan, 3 grains from panicles of different plants of each variety were randomly selected at 14 and 26 DPE and sectioned. Two to 3 images capturing the entire grain were taken, and the areas of endosperm cells were analyzed. A nonparametric Mann–Whitney *U* test determined that on average, starchy endosperm cells in AC Morgan are significantly bigger than CDC Morrison at both timepoints (*P* < 0.001) (Fig. 2, A to E, Supplemental Data Set 1). That the size difference was evident as early as 14 DPE shortly after cellularization is complete supports the notion that the processes controlling cell size are established early in endosperm development. In addition, at 26 DPE, the aleurone cells of AC Morgan are significantly larger than those in CDC Morrison (Fig. 2F) (*P* < 0.001), indicating differences are maintained through the differentiation phase. We did not perform a statistical analysis on wall thickness in the 2 cultivars, but CDC Morrison grains have noticeably thicker cell walls than AC Morgan which is particularly evident at the aleurone/subaleurone interface in 26 and 32 DPE grains (Fig. 2, G and H, arrowheads). In summary, our data are consistent with the earlier findings of Miller and Fulcher (1994) and suggest that this association of reduced cell size with increased MLG content may be commonly observed among oat varieties.

### Cell wall polysaccharide deposition during oat endosperm development

To follow the deposition of polysaccharides and the changes that occur to cell wall composition across endosperm development (0 to 32 DPE), we applied a range of commercially available polysaccharide antibodies and probes to sectioned grains (summarized in Supplemental Table S4). We have observed the deposition of several polysaccharides at selected timepoints using silver enhanced light microscopy (SELM). Here, an enhancement solution is applied to the antibody-labeled sections which causes silver to deposit at the site of the bound gold particles attached to the secondary antibody. The silver deposits provide the contrast for visualizing the site of primary antibody binding which then infers the location and relative abundance of polysaccharide deposition across the endosperm tissue. On occasions when using SELM alone could not provide the resolution needed for accurate comparisons, we sought further clarification and supporting evidence from immunofluorescence and immuno-transmission electron microscopy (TEM).

The following is a detailed description of the spatiotemporal polysaccharide deposition in oat grain, in order of appearance. As for endosperm development, spatiotemporal observations were made in both oat cultivars and images of CDC Morrison shown unless otherwise stated.

### Callose

**Figure 3.**
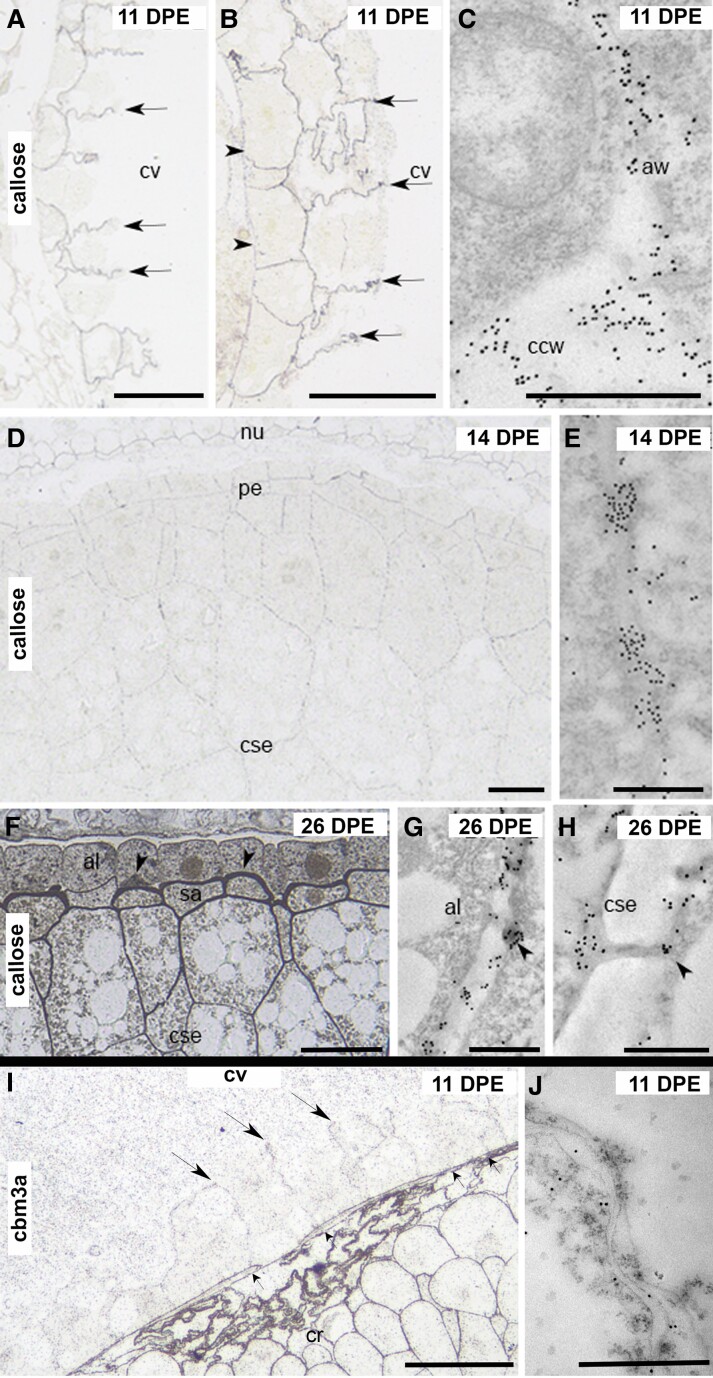
Silver-enhanced light microscopy (SELM) and transmission electron microscopy (TEM) images of (1→3)-β-D-glucan antibody labeling of cell walls during oat endosperm development from 11 to 26 DPE and CBM3a labeling at 11 DPE. **A)** At 11 DPE, callose is present along the early anticlinal cell walls (arrows). **B)** Callose is deposited evenly along the central cell wall (arrowheads) and the early endosperm walls (arrows). **C)** TEM confirms the presence of callose along the central cell wall and the early anticlinal walls. **D)** Once cellularization is complete at 14 DPE, SELM shows that callose deposition has changed from even and continuous to a punctate labeling pattern. **E)** TEM confirms that callose is restricted to regions of plasmodesmata once cellularization is complete. **F)** At 26 DPE, callose labeling is particularly intense at the aleurone/subaleurone interface (arrowheads). **G)** and **H)** At 26 DPE, TEM confirms that callose in the aleurone **G)** and starchy endosperm **H)** is restricted to plasmodesmata (arrowheads). **I)** CBM3a strongly labels the maternal tissues and central cell wall (small arrows) but only lightly labels the early anticlinal walls (large arrows). **J)** TEM confirms that CBM3a labels the early endosperm walls. al, aleurone; aw, anticlinal wall; ccw, central cell wall; cr, crease; cse, central starchy endosperm; cv, central vacuole; nu, nucellus; pe, peripheral endosperm; sa, subaleurone. Scale bar = 100 *μ*m (**A**, **B**, **D**, **F**, and **I**), 1 *μ*m (**C**, **E**, **G**, **H**, and **J**).

(1,3)-β-D-Glucan (callose) is the first polysaccharide to be deposited in the cellularizing oat grain. The first-formed anticlinal walls are evenly and consistently labeled with the anticallose antibody (Fig. 3, A and B, arrows, and C) including the central cell wall (Fig. 3B, arrowheads) from which these early walls emerge. This pattern persists throughout the cellularization phase, and callose is present along the entire lengths of all the early walls that compartmentalize the syncytium into the multicellular endosperm. Soon after cellularization, the pattern of callose deposition changes from a uniform distribution to a punctate pattern reminiscent of the labeling seen in the surrounding maternal tissues that serve as a convenient positive control (Fig. 3D). Closer examination with TEM confirms that callose from about 14 DPE becomes restricted to electron-dense regions surrounding plasmodesmata, plasmodesmatal collars (Fig. 3E), as is observed in vegetative cells. During the later stages of endosperm differentiation, it became increasingly difficult to determine the pattern of callose deposition using SELM alone because of the high number of plasmodesmata in the cell walls of the starchy endosperm and aleurone as the grain ages. However, immuno-TEM clearly shows that from 14 DPE onwards, callose is restricted to only those regions of cell wall containing plasmodesmata (Fig. 3, G and H, arrowheads) except for some intense deposits of callose in the cell walls at the subaleurone/aleurone interface at 26 DPE (Fig. 3F, arrowheads). Callose labeling appears only as punctate deposits in the maternal tissues which correspond to the polysaccharide's restricted location to plasmodesmatal collars in mature cell walls and confirmed by our TEM observations of the pericarp (Supplemental Fig. S3, A and B).

To confirm the labeling patterns described above and to ensure the specificity of the callose antibody, we either preincubated the antibody with 1 mg/mL laminarin ((1,3)-β-D-glucan) prior to application to sections or incubated sections with an endo-(1,3)-β-glucanase. No callose labeling was present in sections pretreated in either manner (Supplemental Figs. S4, A and B, and S5, A and B).

### Cellulose

Detecting cellulose in developing cereal grains is challenging because the probes designed for labeling cellulose also bind to other polysaccharides, such as xyloglucan (XG) and MLG that also contain stretches of the (1,4)-β-D-oligoglucosides recognized by these probes (Wilson et al. 2006; Anderson et al. 2009; Hernandez-Gomez et al. 2015). Given this complexity that could lead to erroneous interpretations, we focused on the very early stages of endosperm development to see if cellulose was present along with callose in the central cell wall and first anticlinal cell walls. The Direct Red 23 dye is known to stain cellulose fibrils with high specificity (Anderson et al. 2009; Liesche et al. 2013), but it did not detect cellulose in early endosperm cell walls.

Next, we applied the XG-specific LM15 antibody (Marcus et al. 2008) to 11 DPE grain sections and discovered no XG labeling in the early endosperm walls. Given this result and knowing that MLG is deposited later in development (see below), we concluded that any labeling with the CBM3a probe at this time was likely cellulose. Labeling with CBM3a was seen along the central cell wall and along the early anticlinal cell walls during cellularization (Fig. 3I, arrows) and confirmed by TEM observations (Fig. 3J). The maternal tissues served as a reliable positive control, and heavy labeling was detected with SELM and TEM, likely due to the abundant levels of MLG in the pericarp (Supplemental Fig. S3, C and D).

### Heteromannan

**Figure 4.**
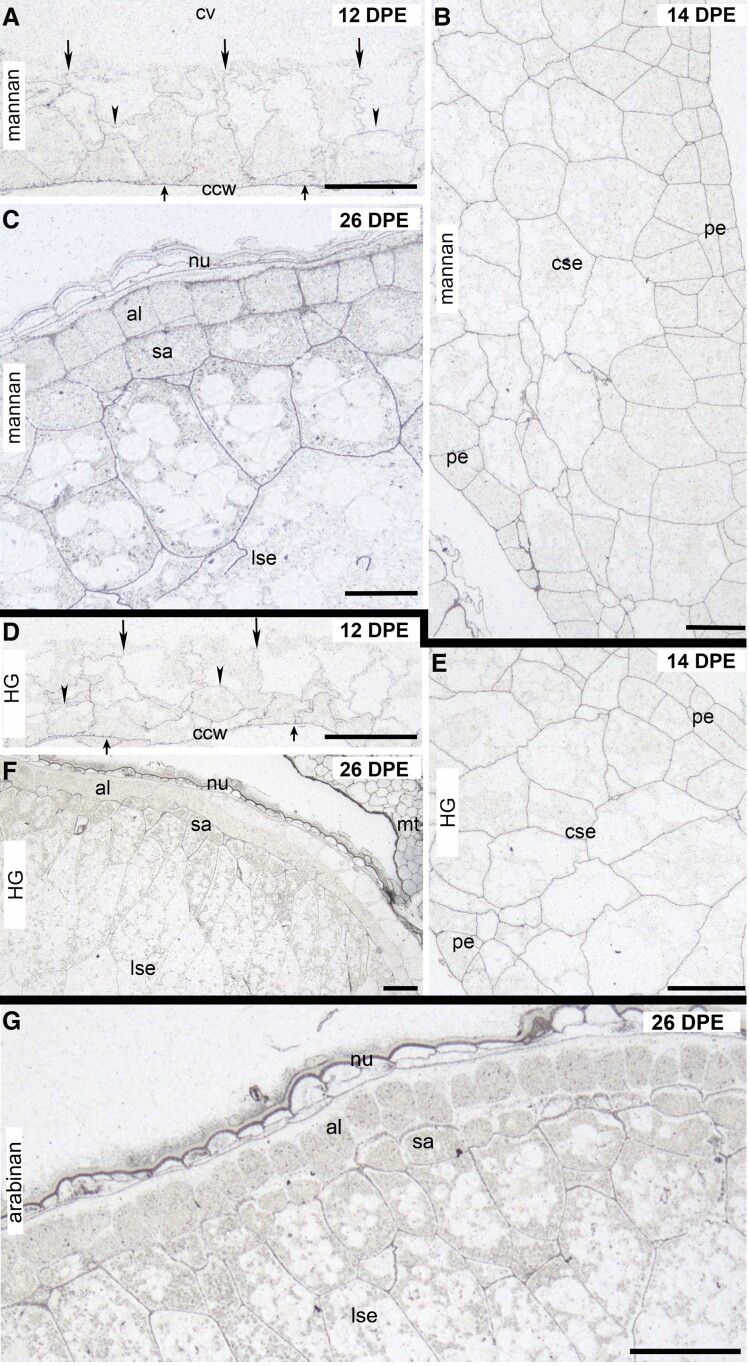
Silver-enhanced light microscopy (SELM) images of HM, HG and arabinan antibody labeling of cell walls during oat endosperm development from 12 to 26 DPE. **A)** HM is present along the central cell wall (small arrows), anticlinal (large arrows), and periclinal (arrowheads) endosperm walls at 12 DPE. **B)** At 14 DPE, HM labeling is strong and very evenly distributed across the peripheral and central endosperm cell walls. **C)** At 26 DPE, HM continues to be distributed throughout the endosperm in very even intensities. **D)** At 12 DPE, JIM7 labels the central cell wall (small arrows), anticlinal (large arrows), and periclinal (arrowheads) endosperm walls (large arrows). **E)** At 14 DPE, JIM7 labels the peripheral and central endosperm walls evenly. **F)** At 26 DPE, JIM7 labels the starchy endosperm and subaleurone cell walls evenly, but the aleurone is unlabeled. The maternal tissues are more intensely labeled than the endosperm cell walls. **G)** At 26 DPE, arabinan can be detected at low but even levels across the starchy endosperm, but labeling is absent from the walls of the aleurone. The remnant nucellar tissue adheres to the aleurone layer and its walls are strongly labeled. al, aleurone; ccw, central cell wall; cse, central starchy endosperm; cv, central vacuole; lse, starchy endosperm cells in lobes; nu, nucellus; pe, peripheral endosperm; sa, subaleurone. Scale bars = 50 *μ*m.

Heteromannan (HM) was first deposited in the endosperm during the cellularization phase at ∼12 DPE (Fig. 4). It was absent from the initial “free-growing” anticlinal walls at 11 DPE and appeared after the first periclinal wall was deposited between daughter nuclei. At the time of deposition, HM was distributed evenly along the central cell wall (Fig. 4A, small arrows) and throughout the early endosperm walls (Fig. 4A, large arrows). At 14 DPE, stronger but very consistent levels of labeling were observed throughout the peripheral and central starchy endosperm (Fig. 4B) and this pattern of labeling persisted through to maturation. At 26 DPE, labeling of equal intensities was observed in the walls of the aleurone, subaleurone, prismatic, and polygonal starchy endosperm cells (Fig. 4C).

### Pectin

Monosaccharide linkage analysis suggested that small amounts of pectin may be present in developing oat grain (Table 2, Supplemental Tables S2 and S3). We attempted labeling with both the LM5 and LM6 antibodies which detect the neutral pectins (1,4)-β-D-galactan (Jones et al. 1997) and (1,5)-α-L-arabinan, respectively (Willats et al. 1998). Weak labeling was only observed with LM6 (Fig. 4G). Despite no homogalacturonan (HG) being detected chemically in mature oat endosperm walls, we tested the JIM7 antibody that recognizes methylesterified HG (Clausen et al. 2003) in an attempt to better visualize the location of pectic polysaccharides in oat grain cell walls. At 12 DPE, JIM7 evenly labeled the early walls of the cellularizing grain but, like HM, HG was not present in the first-formed anticlinal cell walls; rather, it was deposited after the first periclinal division. The JIM7 antibody–labeled along the central cell wall (Fig. 4D, small arrows) and throughout the early endosperm cell walls (Fig. 4D, large arrows) in equal intensities. Between 14 and 26 DPE, HG is present uniformly throughout the peripheral and central starchy endosperm (Fig. 4, E and F). However, at 32 DPE, there was almost no labeling in the aleurone layer and very uniform light labeling across all other regions of the endosperm. The most intense labeling was seen in the maternal tissues and at no point did the labeling in the endosperm reach the intensities observed in the surrounding integuments and pericarp (Fig. 4F). The LM19 antibody, which recognizes partially methylesterified/unesterified HG (Verhertbruggen et al. 2009), was also applied to sections of oat grain across development, but the polysaccharide was only detected in the surrounding maternal tissues including the remnants of the nucellus.

### HX

**Figure 5.**
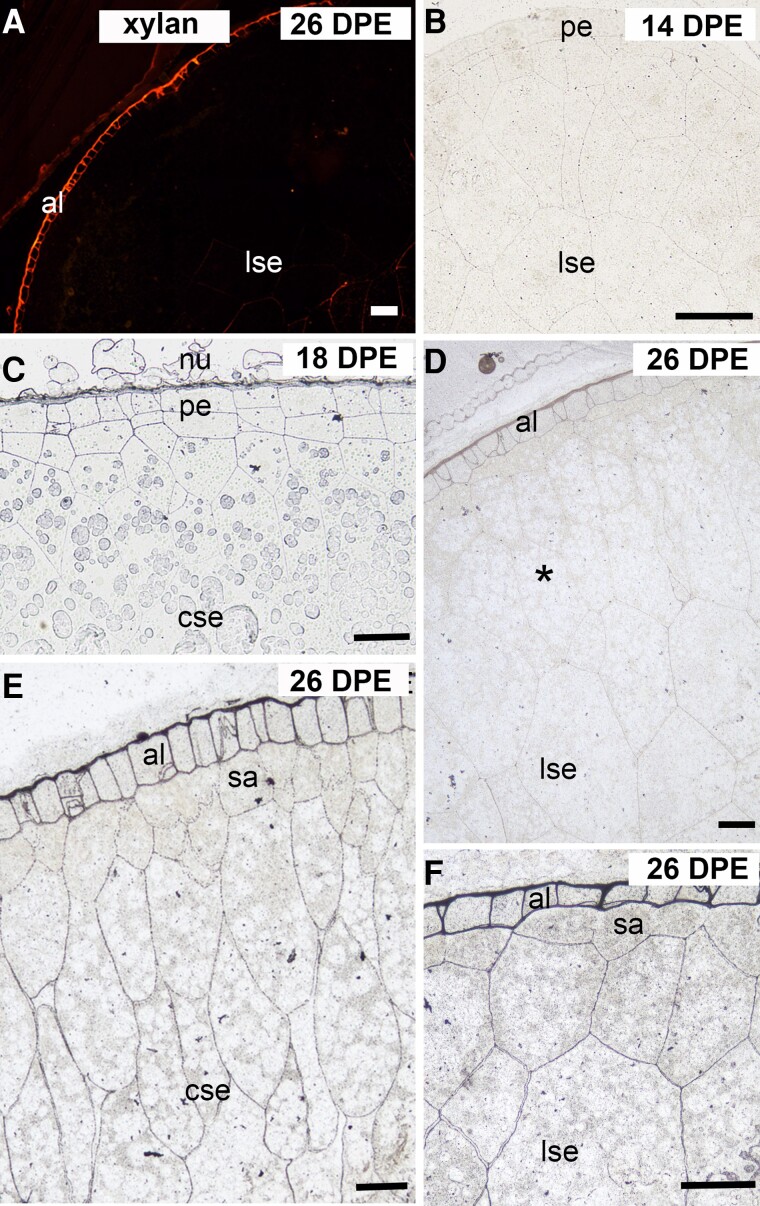
Immunofluorescence and silver-enhanced light microscopy (SELM) of HX antibody labeling of cell walls from 14 to 26 DPE. **A)** At 26 DPE when LM11 is applied alone, only the aleurone layer is labeled, while all other regions of the endosperm are unlabeled. **B)** Pretreating 14 DPE sections with arabinofuranosidase before LM11 reveals low level and even labeling across the peripheral and central endosperm. **C)** At 18 DPE, and only after pretreating sections with arabinofuranosidase, LM11 labels the 2 to 3 cell layers of peripheral endosperm, but no labeling is observed elsewhere in the endosperm. **D)** At 26 DPE and after pretreatment with arabinofuranosidase, labeling in the aleurone is intensified and the starchy endosperm cells located in the lobes previously void of labeling are now labeled. However, the 3 to 4 cell layers beneath aleurone including the subaleurone are still unlabeled. **E)** At 26 DPE and after pretreating sections with NaOH, LM11 antibody labels all areas of the central endosperm including the 3 to 4 cell layers beneath the aleurone. **F)** At 26 DPE, NaOH pretreatment enhances LM11 labeling in all endosperm cells located in the lobes. al, aleurone; cse, central starchy endosperm; cse, central starchy endosperm; lse, starchy endosperm in lobes; nu, nucellus; sa, subaleurone; pe, peripheral endosperm. Scale bars = 50 *μ*m.

We applied several of the commercially available HX antibodies to endosperm sections, including LM28 which detects GlcA-substituted xylan (Cornuault et al. 2015), but had most success with the LM11 antibody aligning this study with our other published reports of HX deposition during barley endosperm development (Wilson et al. 2006, 2012). However, our first attempts to locate (G)AX were unsuccessful; labeling was absent during cellularization and the early differentiation phases. (G)AX labeling was first seen in the aleurone cell walls of 26 DPE grain in addition to small areas of the surrounding maternal tissue but was absent in the starchy endosperm cells located in the central and lobe regions (Fig. 5A, Supplemental Fig. S3, E and F).

Given that (G)AX is the second most abundant polysaccharide in oat endosperm walls (Table 2, Supplemental Tables S3 and S4) and that (G)AX is deposited during the cellularization phase in both wheat and barley (Philippe et al. 2006; Wilson et al. 2012), it seemed unlikely that it would first appear in aleurone cells late in development. Labeling was also weak in maternal tissues; hence, we suspected that HX was present but the backbone epitopes detectable by LM11 (McCartney et al. 2005) were in some way inaccessible. In barley, (G)AX is deposited early in grain development but in a highly substituted (arabinosylated [AX]) form that restricts LM11 binding (Wilson et al. 2012). To determine if, like barley, a highly substituted form of HX is deposited in oat endosperm earlier than 26 DPE, sections were pretreated with α-L-arabinofuranosidase to remove arabinose substituents. HX was absent during cellularization, but soon after, at 14 DPE, we observed very light but even and uniform labeling pattern across all regions of the endosperm (Fig. 5B).

At 18 DPE and only after pretreatment with arabinofuranosidase, LM11 labeled the 2 to 4 cell layers at the periphery of the endosperm but there was very little labeling elsewhere in the endosperm (Fig. 5C). At 26 DPE, HX labeling in the aleurone layer was intensified in sections that had been pretreated with arabinofuranosidase and light labeling was present in the walls of the polygonal cells located within the lobes (Fig. 5D). However, there were several cell layers between the aleurone and the labeled polygonal cells that were either devoid of labeling or had very low levels of labeling (asterisk). A similar labeling pattern was observed in barley when the LM11 antibody was applied without pretreatment (Wilson et al. 2012). This then led us to pretreat sections with NaOH to determine if the HX in these walls was acetylated thus blocking LM11 binding. After pretreating oat grain sections with NaOH, we observed very strong labeling in the walls of the cell layers immediately below the aleurone previously devoid of labeling and an increased intensity of labeling across the entire endosperm (Fig. 5, E and F). Control sections were either labeled with LM11 preincubated with wheat AX or pretreated with xylanase. No labeling was present with either pretreatment even after either arabinofuranosidase or NaOH incubations (Supplemental Figs. S4, C and D, and S5, C and D).

### MLG

**Figure 6.**
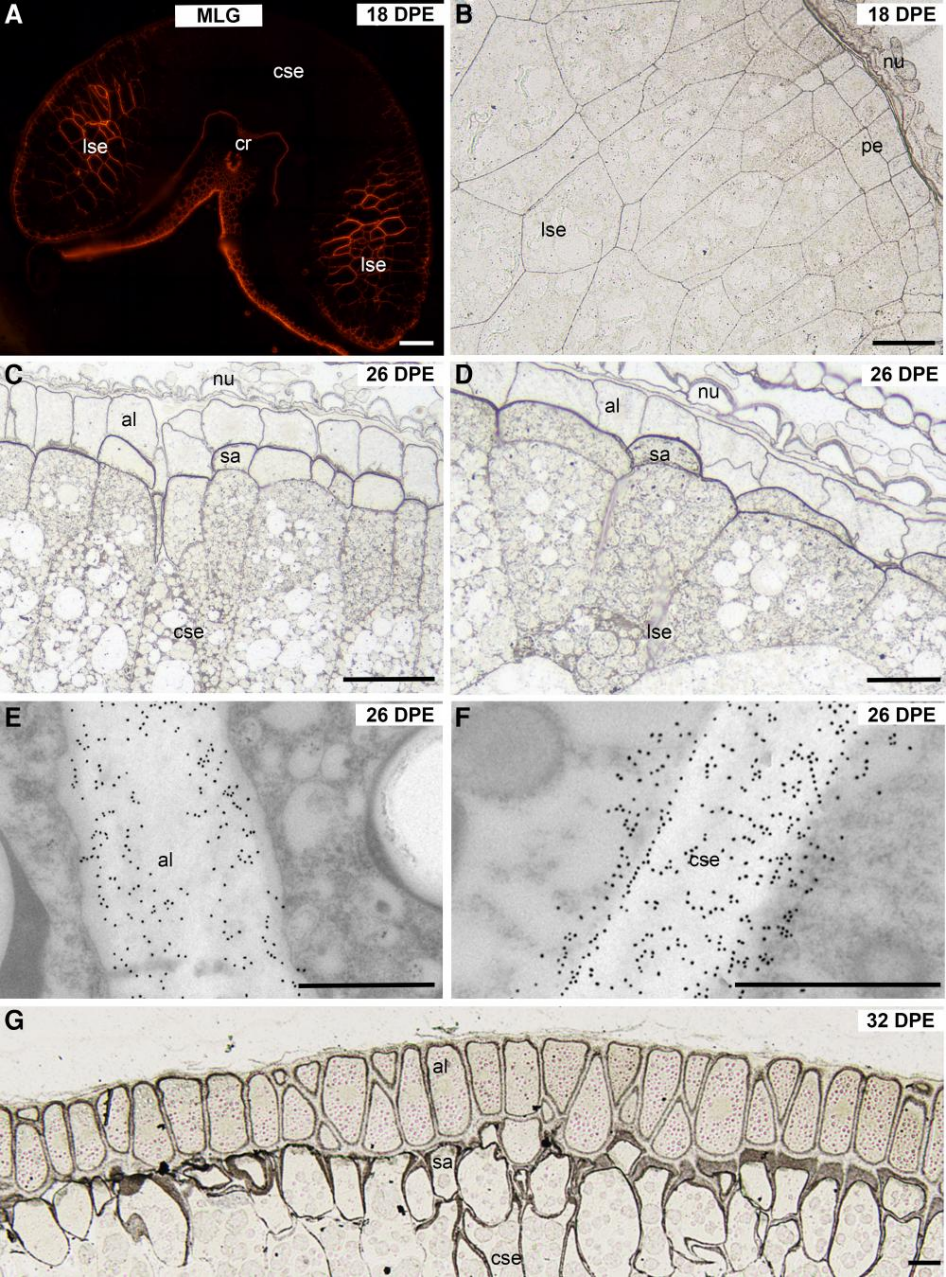
Immunofluorescence, silver-enhanced light microscopy (SELM), and transmission electron microscopy (TEM) images of MLG antibody labeling of cell walls during oat endosperm development from 18 to 32 DPE. **A)** At 18 DPE, MLG is present in the lobes but is absent from the central starchy endosperm walls. **B)** At 18 DPE, the walls of the peripheral endosperm and the starchy endosperm cells located in the lobes are evenly labeled. **C)** At 26 DPE, the central endosperm is now labeled, but the strongest labeling is present in the subaleurone periclinal wall that faces the aleurone. **D)** At 26 DPE, all endosperm walls located in the lobes are well labeled, but the strongest labeling is present at the subaleurone/aleurone interface. **E)** and **F)**. TEM images show that the aleurone **E)** is well labeled, but immunogold labeling is stronger in the starchy endosperm cell walls **F)**. **G)** At 32 DPE, MLG labeling is present in all endosperm walls, but the levels of labeling are highest at the aleurone/subaleurone interface. CDC Morrison grain shown in all images. al, aleurone; cse, central starchy endosperm; pe, peripheral endosperm; al, aleurone; nu, nucellus; sa, subaleurone; lse, starchy endosperm in lobes; cse, central starchy endosperm; cr, crease. Scale bar = 50 *μ*m (**A**, **B**, **C**, **D**, and **G**), 1 *μ*m (**E** and **F**).

Surprisingly, MLG, the most abundant wall polysaccharide of the mature endosperm, is the last polysaccharide to be deposited in the developing oat grain. It was only first detected at 18 DPE, mid-way through the differentiation phase, later than in other cereal grains such as wheat (Philippe et al. 2006), barley (Wilson et al. 2006), and rice (Brown et al. 1997; Palmer et al. 2015). Grains collected at 18 DPE were at various stages of MLG deposition with some having no detectable MLG and others with labeling restricted to the lobes (Fig. 6A). The maternal tissues served as a reliable internal positive control confirming our observations that MLG was absent from earlier stages (Supplemental Fig. S4E). During the cellularization and early differentiation phases, the pericarp and integuments were heavily labeled, while the endosperm cell walls and surrounding nucellus remained unlabeled (Supplemental Fig. S3, G and H).

Initially, labeling was restricted to the differentiating peripheral endosperm and the starchy endosperm cell walls contained within the lobes (Fig. 6, A and B). The central region of grain tissue that connects the 2 lobes was devoid of labeling (Fig. 6A). At 26 DPE, MLG is distributed across the entire endosperm, but the strongest labeling was seen in the walls of the subaleurone, particularly those walls facing the aleurone (Fig. 6, C and D). Our immuno-TEM observations show that MLG deposition in the aleurone layer is confined to the wall while labeling in the starchy endosperm spills over outside of the wall (compare Fig. 6, E and F). At 32 DPE, all regions of the grain are heavily labeled with the MLG antibody. However, SELM observations clearly show that the most intense deposition occurs at the aleurone/subaleurone interface (Fig. 6G). Control sections that were either labeled with MLG antibody pretreated with barley flour or preincubated with lichenase showed no MLG labeling (Supplemental Figs. S4, E and F, and S5, E and F, respectively).

### Comparison of MLG deposition in CDC Morrison vs AC Morgan

**Figure 7.**
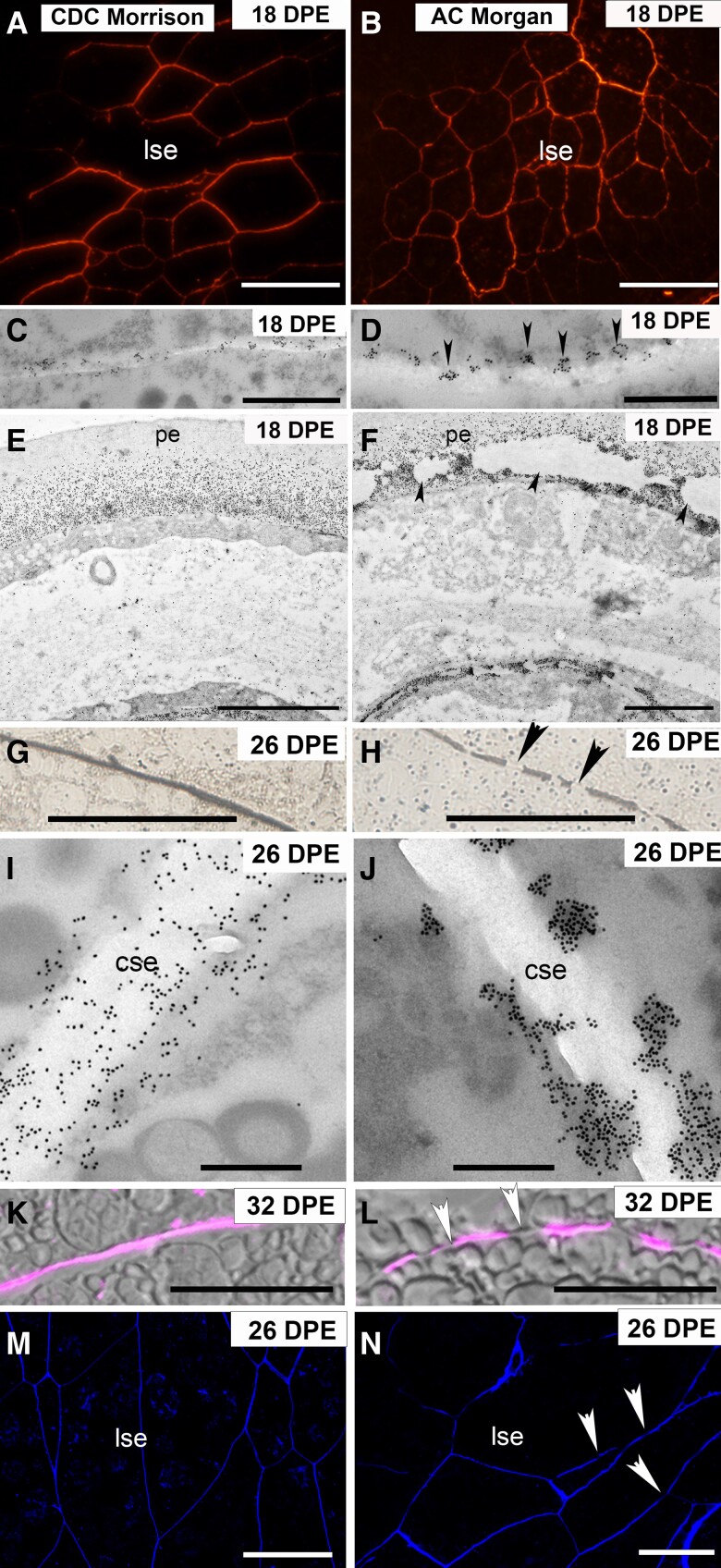
Immunofluorescence, silver-enhanced light microscopy (SELM), and TEM images of MLG antibody labeling of cell walls in AC Morgan and CDC Morrison. **A)** At 18 DPE, immunofluorescence images of MLG labeling in CDC Morrison show an even labeling pattern across the starchy endosperm walls located in the lobes. **B)** In AC Morgan at 18 DPE, the labeling pattern is discontinuous with patches of wall with little to no labeling. **C)** Immunogold labeling confirms that MLG in CDC Morrison is deposited evenly along the starchy endosperm walls, while in **D)** AC Morgan, the MLG is deposited as small electron-dense aggregates positioned at regular intervals along the walls. **E)** TEM reveals that at 18 DPE in CDC Morrison endosperm, MLG is deposited evenly across the peripheral endosperm walls. **F)** At 18 DPE in AC Morgan, there are large areas of wall completely devoid of labeling (arrowheads). **G)** At 26 DPE, SELM of starchy endosperm shows that MLG is present evenly throughout the walls, while in AC Morgan **H)**, there are stretches of wall with little to no labeling. **I)** TEM images of MLG antibody labeling showing an intense but regular deposition of MLG in CDC Morrison, while in AC Morgan **J)**, MLG is present as electron-dense clumps irregularly spaced along the wall. **K)** and **L)** Fluorescence images of MLG labeling in 32 DPE starchy endosperm cells show an even deposition of MLG in CDC Morrison **K)** and a discontinuous pattern in AC Morgan **L)**. Arrowheads indicate regions of wall with no labeling. **M)** and **N)** Calcofluor White staining of polygonal starchy endosperm cells. An even staining pattern is observed in CDC Morrison walls **M)**, while in AC Morgan **N)**, there are unstained regions of wall (arrowheads). cse, central starchy endosperm; lse, starchy endosperm cells located in the lobes; pe, peripheral endosperm; sa, subaleurone. Scale bar = 50 *μ*m (**A**, **B**, **M**, and **N**), 1 *μ*m (**C**, **D**, **I**, and **J**), 2 *μ*m (**E** and **F**), and 10 *μ*m (**G**, **H**, **K**, and **L**).

Unexpectedly, altered patterns of MLG deposition were observed when comparing CDC Morrison to AC Morgan. Immunofluorescence labeling of 18 DPE sections showed the starchy endosperm walls in CDC Morrison are labeled uniformly across the lobe regions, while these same cells in AC Morgan had an uneven or “discontinuous” deposition pattern (compare Fig. 7, A and B). Some areas of the wall are well labeled, but these are interspersed by regions of wall with little to no labeling. Upon further investigation with TEM, we focused on those regions of wall, close to the unlabeled central region that were very lightly labeled, to determine if the different labeling patterns could be identified when MLG is first deposited. MLG labeling was seen as uniform in CDC Morrison with an even distribution of colloidal gold deposited along the wall, while in AC Morgan, small clusters of colloidal gold were positioned in regular intervals along the plasma membrane (PM)–cell wall interface (Fig. 7, C and D). Further investigation of the intensely labeled lobe region in AC Morgan revealed large areas of wall completely devoid of labeling (Fig. 7F, arrowheads) immediately adjacent to areas of heavily labeled wall. This pattern of deposition was never observed in 18 DPE CDC Morrison endosperm where the MLG labeling was consistently heavy across walls located within the lobes (Fig. 7E).

At 26 DPE, a similar pattern of labeling was observed. SELM clearly showed a uniform deposition of MLG in CDC Morrison (Fig. 7G), while in AC Morgan, there were intermittent stretches of wall with little to no labeling (Fig. 7H). Immuno-TEM analysis revealed a very intense and even pattern of labeling in CDC Morrison (Fig. 7I), while in AC Morgan, MLG was detected in large clusters of electron-dense material irregularly positioned along the wall and at the interface with the PM (Fig. 7J). These observations were consistent across the stages of grain development from 18 DPE (Fig. 7, D and F) through to 26 DPE (Fig. 7J). This uneven pattern of MLG labeling was never detected in CDC Morrison but occurred in all regions of the starchy endosperm of AC Morgan with the exception of the aleurone and maternal tissues. We were able to confirm through immunofluorescence labeling that the discontinuous pattern of MLG deposition extended through to grain maturation at 32 DPE in AC Morgan but not CDC Morrison grains (Fig. 7, K and L).

To the best of our knowledge, the discontinuous MLG deposition pattern has not been described elsewhere in any cereal grain or vegetative tissue of plant (or other) origin. It was therefore important to verify that this unexpected pattern was not an artifact introduced by chemical fixation and/or tissue processing. This methodology has been successfully applied to other cereal species in similar immunolabeling studies with no reports of suboptimal cell wall preservation. There were no visible signs of damage or indications of poor fixation in AC Morgan endosperm walls at the timepoints sampled and no discernible differences between unlabeled CDC Morrison and AC Morgan endosperm walls at either low or very high magnifications.

**Figure 8.**
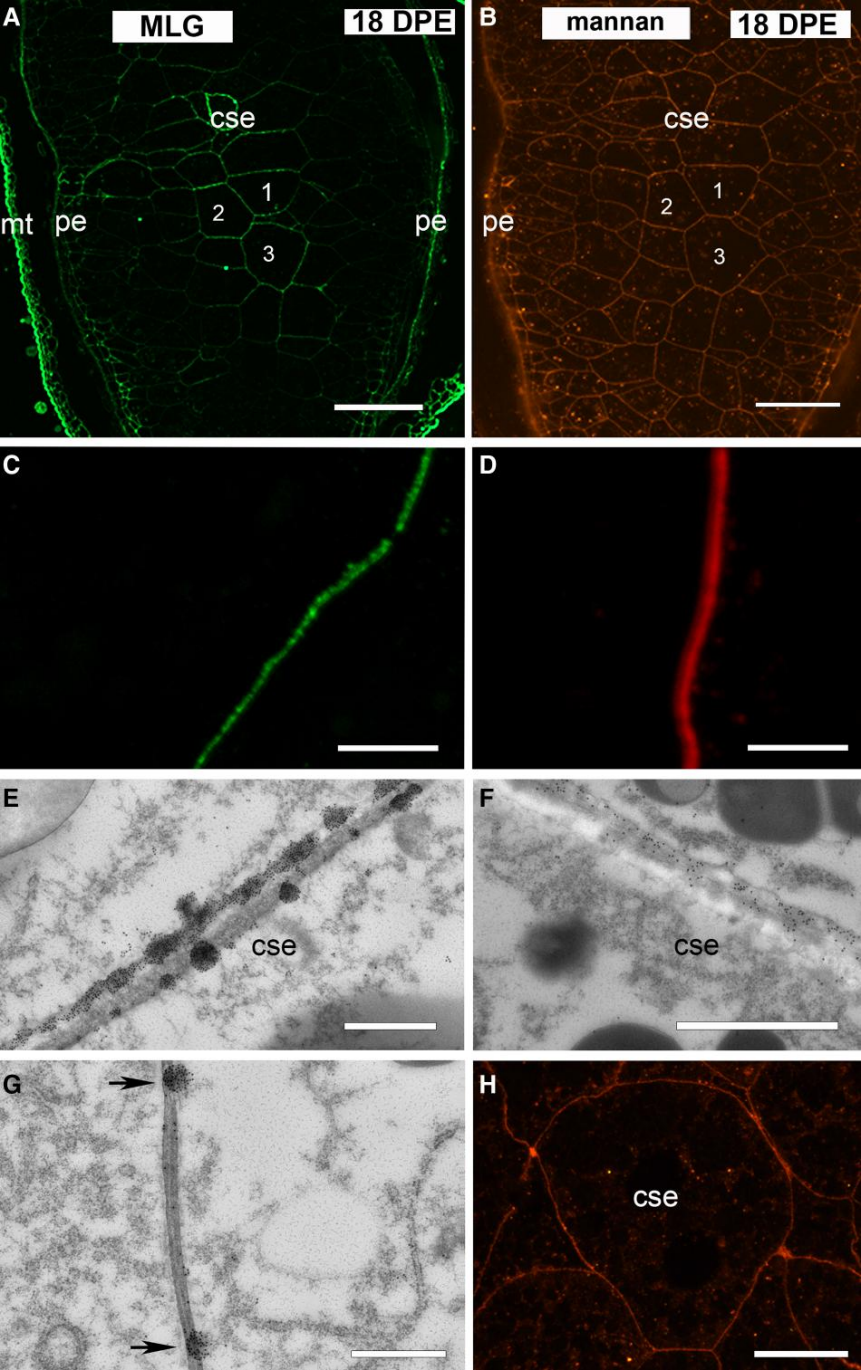
Immunofluorescence and transmission electron microscopy (TEM) images of MLG and HM antibody–labeled serial sections from 18 DPE AC Morgan endosperm. **A)** MLG-labeled 18 DPE section showing an uneven patchy fluorescent labeling pattern along the starchy endosperm cell walls. For example, see cells 1, 2, and 3. **B)** HM-labeled serial section showing an even, consistent fluorescent labeling pattern along the walls in the same region of endosperm (compare walls of cells 1, 2, and 3 with same in **A**). **C)** High-magnification image of MLG-labeled cell wall showing an uneven, patchy fluorescence. **D)** High-magnification image of HM-labeled cell wall showing an even and continuous fluorescent labeling pattern. **E)** TEM image of a MLG-labeled section showing clusters of colloidal gold irregularly positioned along the cell wall. **F)** TEM image showing an even distribution of the HM antibody throughout the starchy endosperm cell wall. **G)** TEM image of MLG antibody–labeled endosperm cell wall showing a >1 *μ*m distance between deposits of MLG (arrows). **H)** Confocal microscope image of anti-H^+^-ATPase fluorescently labeled PM in starchy endosperm cells. cse, central starchy endosperm; mt, maternal tissues; pe, peripheral endosperm. Scale bar = 100 *μ*m (**A** and **B**), 25 *μ*m (**C** and **D**), 1 *μ*m (**E**, **F**, and **G**), 20 *μ*m (**G**).

We therefore proceeded to confirm that the areas of wall that were unlabeled by the MLG antibody held wall contents that could be partly characterized by the binding of another polysaccharide antibody. Unfortunately, both the HX and MLG antibodies are mouse-generated making double or colabeling on the same section difficult, so we instead labeled MLG and HM on alternate serial sections taken from fixed 18 DPE AC Morgan grain. The mannan antibody was the most suitable choice for comparison with MLG labeling because even though chemical analyses showed that there are very low levels of HM in oat endosperm (Table 2), its high avidity causes strong labeling of similar in intensity to the MLG antibody. Confocal microscopy coupled with immunofluorescence revealed a patchy discontinuous deposition of MLG in the central starchy endosperm walls (Fig. 8, A and C), while the HM antibody–labeled these same walls in an even and consistent manner (Fig. 8, B and D). Serial TEM sections were also labeled alternately with the MLG and HM antibodies and confirmed our confocal observations that these probes label the same wall intervals but have vastly different labeling patterns. MLG-labeled sections showed large clusters of colloidal gold irregularly spaced along the wall as well as areas completely void of labeling of more than 1 *μ*m between deposits giving the appearance of a discontinuous deposition pattern at lower magnification (Fig. 8, E and G). Cell walls from the same region of endosperm but labeled with the HM antibody displayed much more uniform labeling (Fig. 8F), indicating these walls in AC Morgan grain are complete. The uninterrupted nature of the signal from anti-H^+^-ATPase, the PM integral protein and marker, within the same cells (Fig. 8H) further supports the view that oat grain tissue is well preserved and cellular integrity is retained during tissue processing for microscopy and cannot explain the discontinuous pattern of MLG labeling in AC Morgan. Together, these observations confirm that the irregular arrangement of MLG identified in this study is real and distinct among the other polysaccharides found in oat endosperm walls. This suggests that there is variation in the MLG deposition process within oat varieties and possibly more broadly within the monophyletic grouping of the commelinid monocots.

After confirming that the discontinuous pattern of MLG deposition in AC Morgan was consistently observed across several biological replicates, we examined whether the pattern was discernible when Calcofluor White was applied to oat grain sections. This fluorescent dye is widely used to stain cell walls and has been shown to bind β-glucans, in particular (Anderson et al. 2009). While it is more difficult to observe the discontinuous pattern in AC Morgan with Calcofluor White staining, it is recognizable, as is the difference in cell size (compare Fig. 7, M and N; Supplemental Fig. S1, F and H, with E and G). Calcofluor White could therefore be a useful tool in future studies of grain morphology, acting as a proxy for detecting the spatiotemporal deposition pattern of MLG.

### Gene expression analysis

**Figure 9.**
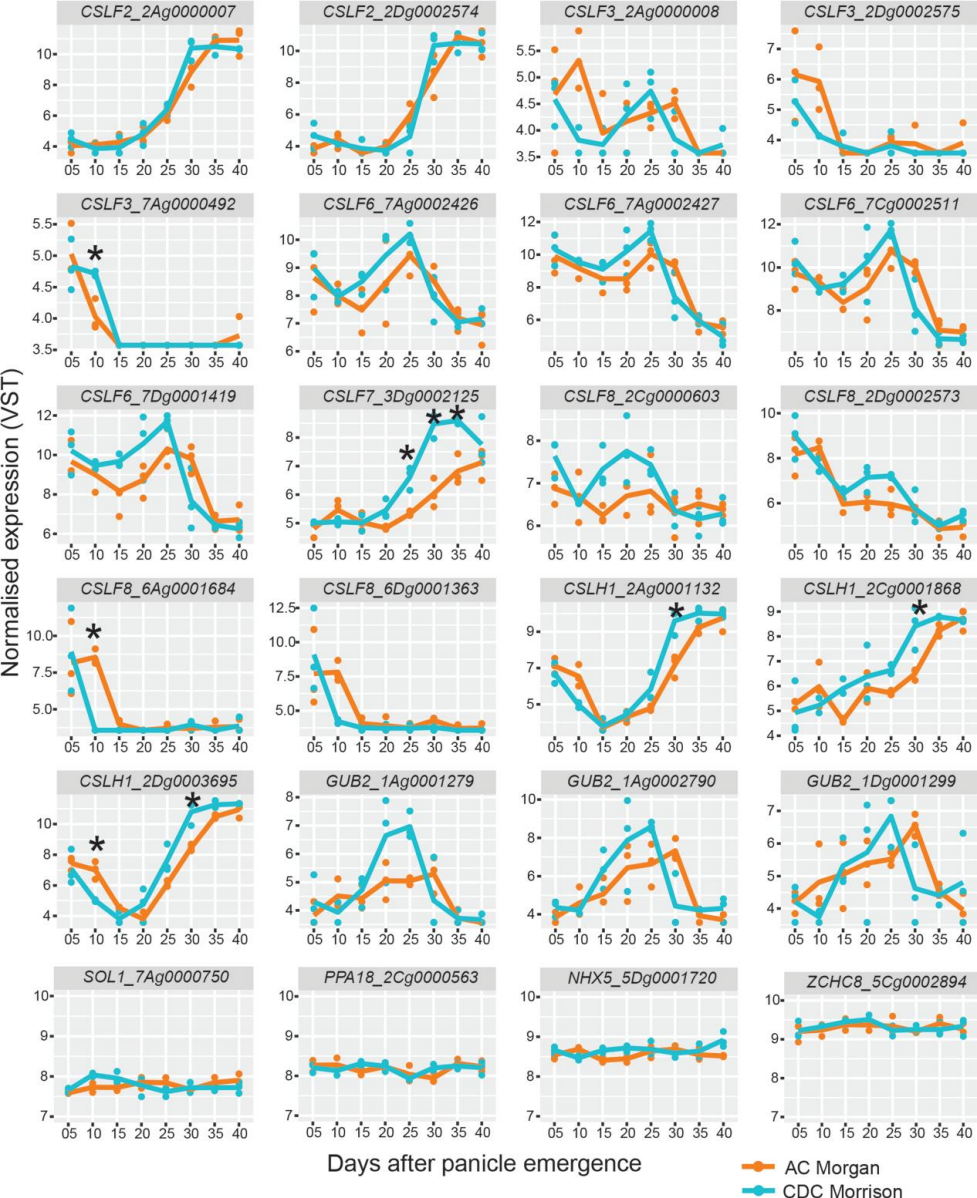
Expression profiles of *CSLF*, *CSLH*, and *GUB* genes during oat grain development in AC Morgan and CDC Morrison. RNA-seq was performed on whole developing grains from 5 to 40 d after panicle emergence. Count data were normalized using VST. Genes annotated as *CSLF*, *CSLH*, and *GUB* (lichenase) are shown. The bottom panel shows 4 control genes whose expression pattern does not change during grain development and is similar between the 2 cultivars. *indicates significant difference (Benjamini–Hochberg adjusted *P* < 0.05) between the 2 cultivars using 2-way ANOVA followed by Tukey’s HSD test.

While undertaking the microscopy study, we simultaneously collected CDC Morrison and AC Morgan grain from 5 to 40 DPE at 5 d intervals for RNA-sequencing (RNA-seq) analysis to assess transcript differences between the 2 varieties. Comparison of the expression of MLG biosynthetic *cellulose synthase-like F, H, J* (*CSLF, H, J*) (Burton et al. 2006; Doblin et al. 2009; Little et al. 2018) and degradation ((1,3;1,4)-β-D-glucan endohydrolase, termed *Glb* or *GUB*) (Slakeski and Fincher 1992) genes revealed some differences in transcript levels and/or timing of several *CSLF* and *CSLH* genes (Fig. 9, Supplemental Table S5). In most cases, gene expression was higher in CDC Morrison than AC Morgan. Interestingly, the majority of these genes were expressed at higher levels early in grain development prior to the onset of MLG deposition in endosperm (18 DPE onwards), implying that they are active in the maternal tissues where MLG was shown to accumulate over this time period (Supplemental Fig. S3, C and G). Although the expression level of homeologs of *CSLF6*, the major MLG synthase of cereals, was not statistically different between the cultivars, they exhibited a trend of higher expression in CDC Morrison from 15 to 25 DPE at which time *CSLH1* homeolog expression increased sharply and significantly more in CDC Morrison than AC Morgan (Fig. 9). These data suggest transcript abundance of MLG biosynthetic genes may contribute to a greater accumulation of MLG in mature CDC Morrison grain. Somewhat paradoxically, the expression levels of *GUB2* genes encoding MLG-specific endohydrolases also trend higher in CDC Morrison compared with AC Morgan over the same period of higher *CSLF6* expression.

## Discussion

Endosperm development in angiosperms is a conserved process with 3 strategies to cellularize the endosperm postfertilization described. In the family Poaceae, the nuclear type of development has been consistently demonstrated in cereal species such as barley (Brown et al. 1994), wheat (Mares et al. 1975), and rice (Brown et al. 1996a, 1996b). In oat, we also observed a nuclear type of endosperm development that is characterized by the formation of a coenocytic syncytium, a multinucleated cell formed from multiple nuclear divisions without cytokinesis (Fig. 1, B to D), the distinguishing feature of this type of endosperm development. In line with observations in other cereal grains (Brown and Lemmon 2007), cellularization of the central vacuole in oat was completed by ∼13 DPE, within a period of ∼2 d (Fig. 1E). The cellularization phase in oat appears to be somewhat shorter than in other cereals such as barley (∼3 d) (Wilson et al. 2006), as suggested by this stage proving difficult to capture. This may partly be a consequence of the sampling strategy: emergence was only scored daily, and pistils were not hand-pollinated and sampled every 8 h, unlike our previous study. Cellularization is followed by a differentiation phase that is complete around 26 DPE (Fig. 1G), proceeded by the maturation phase. Similar to wheat (Shewry et al. 2008), a single layer of aleurone is discerned in oat, while barley has a multilayered aleurone (Aubert et al. 2018). The timeline for the other phases of endosperm development in oat is comparable with those reported in wheat (Philippe et al. 2006) and barley (Wilson et al. 2006), corroborating similarity in the fundamental aspects of endosperm development in cereals.

Of the minor polysaccharides present in mature oat endosperm cell walls (callose/cellulose/XG/HM/pectin/AGPs; collectively accounting for ∼10% *w*/*w*; Supplemental Table S3), from a developmental perspective, the most interesting are callose and cellulose which are the first polysaccharides to be detected in the early anticlinal cell walls at 11 DPE (Fig. 3, A to C, I, and J). Similar to earlier reports in barley (Wilson et al. 2006) and wheat (Philippe et al. 2006), callose was first observed uniformly along the length of the early anticlinal cell walls and the central cell wall during cellularization at 11 DPE, a transient feature as callose is restricted to plasmodesmata by 14 DPE (Fig. 3, D and E) . The presence of callose along the entire length of the early anticlinal cell walls formed during alveolation and cellularization is comparable with the phragmoplast of a dividing vegetative plant cell (Hong et al. 2001). In oat, callose is removed from the phragmoplast by the action of endo-β-1,3-glucanase (Martin and Somers 2004) and its presence is then restricted to the plasmodesmata by 18 DPE where it is proposed to mediate cell signaling and communication (De Storme and Geelen 2014). However, the role of callose deposits associated with the walls at the aleurone/subaleurone interface, previously also observed in subaleurone cells of barley endosperm (Fulcher et al. 1977; Wilson and Bacic 2012), remains unclear and is speculated to arise from a mechanical stress response (compression) during the final stages of grain filling (Fulcher et al. 1977; Bacic and Stone 1981a, 1981b; Wilson et al. 2012). Unlike vegetative plant cells where cellulose is the major load-bearing polysaccharide, in endosperm cells, it is present in low abundance as these cells are not exposed to the same mechanical forces and therefore remains at low levels (5% to 10% *w*/*w*) throughout endosperm development. Other minor polysaccharides, such as HM (∼2% *w*/*w*; Supplemental Table S3) and HG (∼1% *w*/*w*; Supplemental Table S3), were deposited early in cellularization around 12 DPE after the first periclinal walls are laid down.

The second most abundant polysaccharide of oat endosperm walls (12.73% and 13.45% *w*/*w* in AC Morgan and CDC Morrison, respectively; Table 2, Supplemental Tables S2 and S3) is (G)AX, and in contrast to barley (Wilson et al. 2012), its deposition coincided with completion of cellularization and was detected from 14 DPE onwards. Surprisingly, it was initially only detected at 26 DPE, that is, late in the differentiation phase, but after performing a complex series of demasking experiments (urea/arabinofuranosidase/NaOH/lichenase; see Results section), its backbone epitopes were revealed to be present at 14 DPE (see Fig. 5). This is consistent with both wheat and barley (Philippe et al. 2006; Wilson et al. 2012) where (G)AX is deposited during the cellularization phase. These demasking experiments suggest a number of possible interpretations which would also impact on wall organization and solubility of the (G)AX in the mature grain: (i) interaction of the (G)AX with other wall polysaccharides (as revealed by increased binding of the LM11 antibody after both swelling of the walls with the chaotropic reagent urea and lichenase digestion to remove the MLG), a well-described phenomenon observed from immunolabeling of plant tissues (e.g. Wilson and Bacic (2012)); (ii) a highly substituted xylan backbone deposited during development, with either glycosyl residues (primarily arabinose) or acetyl residues, as revealed by increased binding of the backbone epitopes detectable by LM11 after arabinofuranosidase and NaOH treatments, respectively; and (iii) a combination of both association with other wall polysaccharides and a high degree of backbone substitution. Interestingly, the degree of glycosyl (arabinose/glucuronic acid) substitution of the xylan backbone in the mature oat walls (Table 2, Supplemental Tables S2 and S3) is not excessively high (∼25%) which could either mean that these glycosyl residues are distributed evenly across the backbone, roughly 1 residue in 4 (which would impede LM11 binding), or that there is heavy acetylation of the xylan backbone during development; since xylans are known to be modified extensively postdeposition (dearabinosylated and deacetylated), either or both could be contributing to this masking.

MLG is a major noncellulosic cell wall polysaccharide of cereal endosperm constituting ∼75% *w*/*w* in barley and 20% *w*/*w* each in wheat and rice (Bacic and Stone 1981a, 1981b; Miller et al. 1995). Deposition of MLG in wheat and barley is known to occur during the cellularization phase (Philippe et al. 2006; Wilson et al. 2006) and the early differentiation phase (Brown et al. 1997; Palmer et al. 2015) of grain development in rice. In contrast, for oat, MLG was observed to be first deposited late in the differentiation phase at 18 DPE (Fig. 6, A and B).

For both AC Morgan and CDC Morrison, heavy labeling of the subaleurone layer with MLG antibody when compared with that of the aleurone was confirmed using all the microscopy modalities and was consistent with earlier findings (Miller and Fulcher 1994). It was also demonstrated that high MLG oat variety Marion had smaller cells and thicker cell walls when compared with a low MLG variety OA516-2 that had a larger cell size and thinner cell walls (Miller and Fulcher 1994). The 2 cultivars examined in this study also followed this trend where AC Morgan is a higher yielding variety (Kibite and Menzies 2001) with a larger endosperm cell size, more starch, and lower MLG content, while CDC Morrison is a lower yielding variety with a smaller cell size, less starch, and a higher MLG content (Table 1, Supplemental Table S1). Indeed, an inverse association between starch and MLG content is commonly observed within Pooideae species (Trafford et al. 2013). In this comparative study of barley and purple false brome (*Brachypodium distachyon*) representing the high and low starch/MLG ratio “extremes,” respectively, the factors responsible for the variation in this ratio were examined. Based on their analysis, the authors propose that a reduction in endosperm cell expansion, and in the case of *B. distachyon*, a reduced ability to synthesize starch, results in a greater amount of carbon available for MLG production in the endosperm. The interdependency of starch and MLG is elegantly demonstrated in cereal mutants. For example, the barley starch synthase IIa mutant, *sex6* (Morell et al. 2003), displays enhanced MLG content concomitant with starch changes in the grain and conversely *B. distachyon cslf6* mutants show reduced MLG levels and increased starch content (Bain et al. 2021). Further examination at the molecular level is needed to determine the mechanism by which AC Morgan endosperm cells have an enhanced capacity to expand and accumulate starch relative to the same cells of CDC Morrison.

Most interestingly, and not observed in previous comparisons of oat varieties, was that the pattern of MLG deposition between the 2 cultivars differed. Endosperm walls in CDC Morrison labeled uniformly, while the same cells in AC Morgan had an uneven or “discontinuous” pattern of deposition (compare left and right columns in Fig. 7). Focusing on the walls with early deposition at 18 DPE, we saw uniform MLG labeling in endosperm walls within the lobes of CDC Morrison (Fig. 7, C and E) yet a strikingly different pattern in AC Morgan. In AC Morgan, we initially observed small clumps of immunogold particles in electron-dense regions near to or at the PM (Figs. 7, D and F, and 8, E and G). Looking later in development, these patches of immunogold labeling within or the near vicinity of the wall increased in size and unlabeled stretches of wall were also revealed. The length of these unlabeled wall regions is estimated to be similar when images obtained with the alternate microscopy modalities are compared, suggesting the “gaps” in MLG label observed later in development (26 DPE and beyond) correspond with the electron-lucent regions observed earlier (compare Fig. 7, H and I, with F and Fig. 8, E and G).

It is currently unknown how these different spatiotemporal patterns of MLG deposition are established but molecular differences clearly exist between the varieties, at least from the initiation of MLG deposition at 18 DPE given distinct patterns of deposition are observable from this time onwards. Some plausible possibilities as to how the MLG deposition differences manifest may be due to variation in the frequency of delivery and/or turnover rate of CSLF6, the major MLG synthase of grasses (Burton et al. 2006; Tonooka et al. 2009; Taketa et al. 2012; Vega-Sánchez et al. 2012; Wilson et al. 2015; Bain et al. 2021), and or subtleties in the mechanism/type of vesicular delivery to the PM (van de Meene et al. 2017). The electron-dense deposits that in most cases accompany MLG deposition in AC Morgan but to a far lesser extent in CDC Morrison suggest their vesicular type/contents are distinct and could indicate some variation in origin and/or transport pathway of vesicles carrying the MLG synthesis machinery from the Golgi apparatus to the PM. Although we do not currently have an explanation for these observations, this could be a consequence of variation in, for example, the process(es) of intracellular vesicular transport of the biosynthetic enzymes arising from subtle differences in CSLF6 primary amino acid sequences. Variances in expression of CslF6 homeologs in hexaploid oat (*A. sativa*) have been reported. For example, high expression of *AsCslF6_A* and *D* was observed in a high MLG cultivar, whereas high expression of *AsCslF6_C* was linked with a low MLG cultivar (Fogarty et al. 2020; Zimmer et al. 2020). In this study, we did not detect major differences in expression of the *AsCslF6* homeologs in CDC Morrison and AC Morgan grain (Fig. 9, Supplemental Table S5), suggesting another mechanism is operating in these cultivars. The observation that expression levels of *GUB2* genes encoding MLG-specific endohydrolases are elevated over the period of higher *CSLF6* expression in CDC Morrison compared with AC Morgan implies that endosperm MLG accumulation can occur under conditions of higher MLG turnover. An alternative explanation may be the MLG-specific endohydrolases play a role in MLG biosynthesis, similar to the function proposed for Korrigan1, the endo(1,4)-β-D-glucanase associated with cellulose production (McFarlane et al. 2014). Further experimental work is required to understand how the spatiotemporal differences in MLG manifest between genotypes.

## Conclusions

This study employed 3 microscopy modalities, SELM, immunofluorescence microscopy, and immuno-TEM, to reveal the morphological details of oat endosperm development and the spatiotemporal patterning of cell wall polymer deposition during this process. SELM was able to provide an overview of labeling across the developing grain and amplify weak immunolabeling without excessive background signal, limitations of TEM, and immunofluorescence microscopy, respectively. In the case of MLG labeling, immunofluorescence microscopy was key to identifying the discontinuous pattern within the endosperm cells of the low MLG content line, AC Morgan. This aided the targeted use of SELM and TEM to explore in detail the spatiotemporal patterning of MLG deposition, uncovering important aspects of this process not possible if only 1 microscopy modality was employed. We also used RNA-seq to reveal the expression patterns of the major MLG biosynthetic (*CSLF, H, J*) and degradative (*GUB*) genes during grain development, and this has revealed that, as for cellulose biosynthesis, MLG assembly and deposition may involve some interplay between synthesis and turnover.

In summary, our findings indicate that the wall architecture of the oat varieties used in this study differ in a more profound way than simply a quantitative difference in MLG content and it is tempting to speculate whether the differences in the pattern of MLG deposition result in differing solubilities/accessibilities of the MLGs between these varieties.

## Materials and methods

### Plant growth, maintenance, and collection

Seed of 2 Canadian oat (*A. sativa*) cultivars AC Morgan and CDC Morrison was sourced from PepsiCo Pty Ltd (Minnesota, USA) and grown in a climate-controlled glasshouse under long day conditions (23 °C 14 h day/19 °C 10 h night). Prior to planting, seeds were treated with commercial fungicide, Baytan T (Bayer, 4 g/L solution) and then 4 seeds were sown in 6-L plastic pots filled with general potting mix (Bio Gro, Australia). Germinated seedlings were thinned to 1 plant per pot, and plants were maintained year-round at a constant temperature of 21 °C in a 12 h dark, 12 h light cycle. Pots were watered daily with drip feeders and a stock of in-house fertilizer solution containing N:P:K = 155:227:285 g/L, and other micronutrients were added weekly. Panicles were dated on the day of heading when the first spikelet of the inflorescence emerged from the flag leaf, defined as 0 DPE. For all microscopy and RNA-seq work, the larger of the 2 developing caryopses within the double kernel spikelet characteristic of both cultivars was harvested from the upper-middle section of the panicle.

### Oat grain fixation

Developing oat grains were collected, and the outer tissues of the floret (glumes, palea, and lemma) were removed. The dissected grains were fixed in 4% *v*/*v* glutaraldehyde (Sigma-Aldrich, #G5882) in 1× phosphate buffered saline (PBS) (3.7 mM NaCl, 2.7 mM KCl, 4.3 mM Na_2_HPO_4_, 1.4 mM KH_2_PO_4_ adjusted to pH 7.2) and placed under gentle vacuum up to 48 h at room temperature (RT) and stored at 4 °C before processing. For each developmental stage, at least 12 grains were collected from each of 3 plants and several collections made over the duration of the study.

### Postfixation and embedding of grain

Fixed grains were processed according to the method described by Wilson and Bacic (2012) using London Resin (LR) White (ProSciTech, Kirwan, Qld, Australia) as the embedding resin.

### Sectioning

For light and confocal microscopy, 1-*μ*m-thick sections were cut from the resin-infiltrated blocks using a Leica Ultramicrotome EM UC7 (Leica Microsystems Pty Ltd, Macquarie Park, NSW, Australia) fitted with a 4-mm histo-diamond knife (ProSciTech). Sections were collected on glass slides and dried on a hotplate set to 135 °C. For TEM, 100-nm sections were cut on an ultra 45° diamond knife and collected on 300 mesh formvar-coated gold grids (ProSciTech).

### Histology

Sections allocated for studying grain architecture were flooded with a 1% *w*/*v* Toluidine Blue O solution containing 2% *w*/*v* borax (Sigma-Aldrich) for a few seconds and then washed in water and mounted in 50% *v*/*v* glycerol for viewing and image capture with an Olympus BX51 upright widefield microscope fitted with a Tucsen digital camera and running Mosaic acquisition software (Olympus, Japan). Sections were separately stained for 15 min in 0.1% *w*/*v* Calcofluor White (Sigma-Aldrich), washed in water, and mounted in 50% *v*/*v* glycerol. To detect cellulose in early endosperm walls, sections were stained in 0.1 mg/mL Direct Red 23 (Sigma-Aldrich) in PBS for 20 min, washed, and mounted in 50% *v*/*v* glycerol. Images of Calcofluor White and Direct Red–stained sections were taken on a Nikon Eclipse Ti fluorescence microscope. Unless otherwise shown, positive interaction with a wall probe was deduced by comparison with the appropriate negative control.

### Immunolabeling

Sections for immuno-light and electron microscopy were pretreated with 2 M urea for 30 min then thoroughly washed in PBS. A blocking buffer containing 1% *w*/*v* BSA in PBS was then applied for 30 min before incubating sections in a dilution of primary antibody in blocking buffer for a minimum of 1 h at RT then 4 °C overnight. The primary antibodies, their substrates, and the concentrations used are summarized in Supplemental Table S4. After washing in PBS to remove primary antibody, sections were treated with blocking buffer for 30 min and incubated for 1 h at RT in a 1:20 dilution of either antirat or antimouse IgG (H+L) 18 nm colloidal gold for TEM (Jackson ImmunoResearch, West Grove, Pennsylvania, USA). Sections used for immunofluorescence microscopy with all cell wall probes except CBM3a were incubated in an Alexa Fluor 488 or 568 donkey antimouse/antirat IgG (H+L) secondary antibody diluted 1:200 in blocking buffer. To detect cellulose, sections were incubated in a 1:50 dilution of CBM3a in PBS for 1 h at RT followed by treatment with a monoclonal antipolyhistidine antibody (Thermo Fisher Scientific, Scoresby, Vic., Australia) diluted in blocking buffer to 1:75 for 1 h at RT. Sections were then washed and incubated in an appropriate antimouse secondary antibody. A rabbit polyclonal H^+^-ATPase (Agrisera, AS07260) was used at a 1:100 dilution in combination with an Alex Fluor 568 antirabbit IgG (H+L) secondary antibody (1:200) for PM labeling.

### SELM

Sections incubated in goat antirat IgG (H+L) ultrasmall gold (average < 0.8 nm) (Aurion, Wageningen, The Netherlands) were washed in PBS and fixed for 15 min in 2.5% *v*/*v* EM grade glutaraldehyde (Sigma-Aldrich) in PBS. Sections were thoroughly washed with water and then incubated for 5 min in a solution containing equal volumes of silver enhancement kit solutions A and B (Sigma-Aldrich) according to the manufacturer's instructions. After washing, the labeled sections were mounted in 50% *v*/*v* glycerol in water and imaged as per Toluidine Blue–stained sections.

### Immunofluorescence microscopy

After secondary fluorescence antibody labeling, the sections were mounted in PBS, covered with a #1.5 coverslip, and imaged on a Leica DM6000B widefield fluorescence microscope equipped with a DFC 450 color CCD camera and EL6000 light source (Leica Microsystems). For the Alexa Fluor 488 antibody labeling, the FITC filter cube was used (Ex BP 450-490, DC 510 nm, Em LP 515), and for the Alexa Fluor 568 antibody labeling, the dsRed filter cube was selected (Ex BP 580/20, DC 510, Em 630/55). Both were imaged with a gain of 3 and exposure time of 1 s. For calcofluor staining, the DAPI filter cube (Ex BP 350/50, DC 400, Em 460/50) was used with a gain of 1 on the camera and an exposure of 300 ms. Brightfield differential interference contrast (DIC) images were acquired with a 100 ms exposure.

### TEM

After secondary antibody incubations, grids were washed in ultra-high-quality water and stained with 2% *w*/*v* uranyl acetate (ProSciTech) for 5 min, washed, and then air dried before carbon-coating using an Emitech K950X instrument (Quorum Technologies, Laughton, East Sussex, UK). TEM was conducted using a Jeol JEM-2100 transmission electron microscope under an accelerating voltage of 80 kV and images captured on a Gatan Orius SC 200 CCD camera (Scitek, Australia).

### Control experiments

To ensure observed labeling was a result of antibody binding and not nonspecific background, 2 kinds of control experiments were performed. Diluted antibodies were either preincubated in their respective substrate/antigen or a linkage-specific enzyme was applied to sections before antibody labeling to remove the target polysaccharide or its substituents. All substrates and enzymes (see Supplemental Table S4) were applied at a concentration of 1 mg/mL in PBS at RT for 1 h. For xylan detection, a solution of 0.05 M NaOH was applied to sections for 90 min at RT in an attempt to remove ester-linked acetyl groups blocking binding to the LM11 antibody.

### Cell area and statistical analysis

Oat grain sections stained with Toluidine Blue were subjected to analysis using “FIJI is just ImageJ,” software (Schindelin et al. 2012). In 6 to 9 images per timepoint for each cultivar, the largest 10 endosperm cells for which the entire cell outline was observable were chosen to calculate the cell area using the polygon selection tool. Statistical analysis was undertaken with SPSS statistics software (version 28.0.1.0, IBM, Sydney, Australia). See Supplemental Data Set 1 file for further details.

### RNA extraction

Developing grains were first collected from fully emerged panicles at 5 DPE and then at 5 d intervals from 5 to 40 DPE. For each of 3 biological replicates, 10 individual grains (glumes removed) from a single panicle were pooled into a 2-mL tube and snap-frozen in liquid nitrogen. Frozen oat grains (100 mg/sample) were ground to fine powder using a plastic pestle. The RNA was extracted using the Spectrum Plant Total RNA Kit (Sigma-Aldrich) with some additional optimization steps. Samples were pretreated with 0.03% *v*/*v* α-amylase (Sigma-Aldrich, A4551-100MG) in lysis buffer, vortexed for 1 min, and incubated at RT for 6 min. β-Mercaptoethanol was then added to 0.01% *v*/*v* and samples vortexed again for 30 s prior to centrifugation at RT for 5 min at ∼16,000 *× g*. The supernatant was then transferred to a filtering column, and the Spectrum Plant Total RNA protocol was followed using the manufacturer's instructions including the on-column DNase treatment. Protocol A was used for the binding buffer solution. Samples were further purified using RNA Clean & Concentrator-5 kit (Zymo Research, R1016) to ensure sufficient RNA purity and concentration. RNA analyses were undertaken using a 4150 TapeStation System (Agilent) with RNA Screen Tapes (Agilent, 5067-5576) following the manufacturer's instructions.

### RNA-seq library construction and sequencing

Libraries were generated using a TruSeq Stranded mRNA Library Prep Kit (Illumina) according to the manufacturer's instructions and sequenced on a NextSeq 500 platform (Illumina) as single-end reads. Read quality was examined using FastQC (http://www.bioinformatics.babraham.ac.uk/projects/fastqc), and low-quality reads and adapters were trimmed using Trim Galore (https://github.com/FelixKrueger/TrimGalore). To remove rRNA, the reads were mapped to the SortMeRNA rRNA database using Bowtie2, and unmapped reads were extracted (Kopylova et al. 2012). To quantify transcript abundances, reads were mapped to the oat transcriptome (OT3098 v2, “Avena sativa—OT3098 v2, PepsiCo, https://wheat.pw.usda.gov/jb? data=/ggds/oat-ot3098v2-pepsico”) using Salmon (Cheng et al. 2017; Patro et al. 2017). Raw gene-level counts were obtained using tximport (Soneson et al. 2016), and genes with raw count <5 in 5% of all samples were excluded from downstream analysis. Raw counts of all 3 biological replicates from all 8 timepoints were normalized using variance-stabilizing transformation (VST) from the DESeq2 package (Love et al. 2014). Two-way ANOVA (genotype and timepoint with interaction) followed by a Tukey's honestly significant difference (HSD) was performed in R (version 4.0.3) on a focused set of known MLG-related transcripts. Variance of all expressed genes was calculated using the VST on normalized data, and the top 4 genes with lowest variance were selected as exemplar stably expressed genes.

### Sequence data availability

The short-read sequence data described in this manuscript can be found in Gene Expression Omnibus (record number: GSE224726). The transcriptome annotation data can be found at GrainGenes (https://wheat.pw.usda.gov/GG3/graingenes-downloads/pepsico-oat-ot3098-v2-files-2021). Genes can be cross-referenced using gene identifiers referred to in Fig. 9 and Supplemental Table S5.

## Supplementary Material

kiad566_Supplementary_Data

## Supplemental data

The following materials are available in the online version of this article.

**Supplemental Figure S1.** Toluidine Blue and Calcofluor White staining for comparison of oat grain endosperm architecture.

**Supplemental Figure S2.** Toluidine Blue staining of sections of CDC Morrison and AC Morgan endosperm at 5, 14, and 26 DPE for developmental comparison.

**Supplemental Figure S3.** Positive antibody controls showing immunolabeled maternal tissues.

**Supplemental Figure S4.** Preabsorption controls.

**Supplemental Figure S5.** Enzyme controls.

**Supplemental Materials and Methods.**

**Supplemental Table S1.** Agronomic and grain quality characteristics of Canadian oat spring varieties CDC Morrison and AC Morgan.

**Supplemental Table S2.** Monosaccharide linkage analysis of “popped” endosperm cell walls of mature grain of CDC Morrison and AC Morgan.

**Supplemental Table S3.** Calculated polysaccharide composition of “popped” endosperm cell walls of mature CDC Morrison and AC Morgan grain.

**Supplemental Table S4.** Cell wall antibody probes, substrates, and cell wall degrading enzymes used in this study.

**Supplemental Table S5.** Expression of *CSLF*, *CSLH*, and *GUB* family genes during oat grain development.

**Supplemental Data Set 1.** Comparison of endosperm cell size in CDC Morrison and AC Morgan grain at 14 and 26 DPE.
