# Supplementary material for: Spatiotemporal deposition of cell wall polysaccharides in oat endosperm during grain development
Source: Plant Physiol. 2023 Oct 20;194(1):168–89. doi: 10.1093/plphys/kiad566 (PMC10756759; doi:10.1093/plphys/kiad566)
Supplement: kiad566_Supplementary_Data [file kiad566_supplementary_data.zip › Nadiminti et al Supplemental Figures Tables X Methods PROOF.pdf]

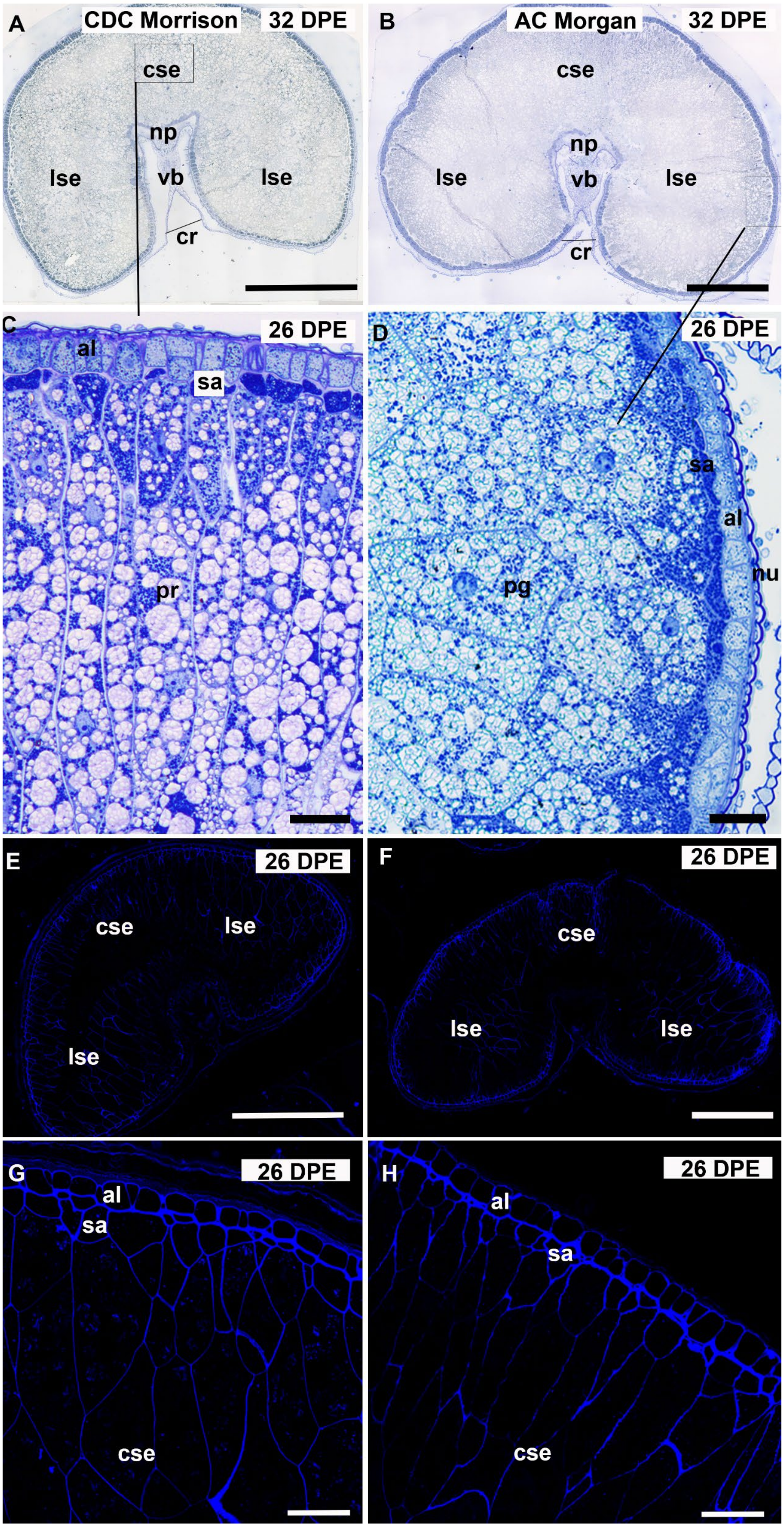

Supplemental Figure S1; Toluidine Blue and Calcofluor White staining for comparison of oat grain endosperm architecture. A. Whole transverse section of CDC Morrison grain at 32 days post-emergence (DPE). **B.** Whole transverse section of AC Morgan grain at 32 DPE. **C.** 26 DPE endosperm showing the central starchy endosperm region of aleurone, sub-aleurone and prismatic starchy endosperm cells. **D.** Image highlighting the polygonal starchy endosperm cells located in the lobes. **E**, **F**. Calcofluor White staining of whole **(E)** CDC Morrison and **(F)** AC Morgan grains. Higher magnification image from the central starchy endosperm of **(G)** CDC Morrison and **(H)** AC Morgan. Note the discontinuous staining in the prismatic cells. al, aleurone; cse, central starchy endosperm; lse; starchy endosperm cells in lobes; pg, polygonal cells; np, nucellar projection; nu, nucellus; pr, prismatic cells; sa, sub-aleurone. Scale bar = 1 mm (A, B, E, F), 50 µm (C, D, G, H).

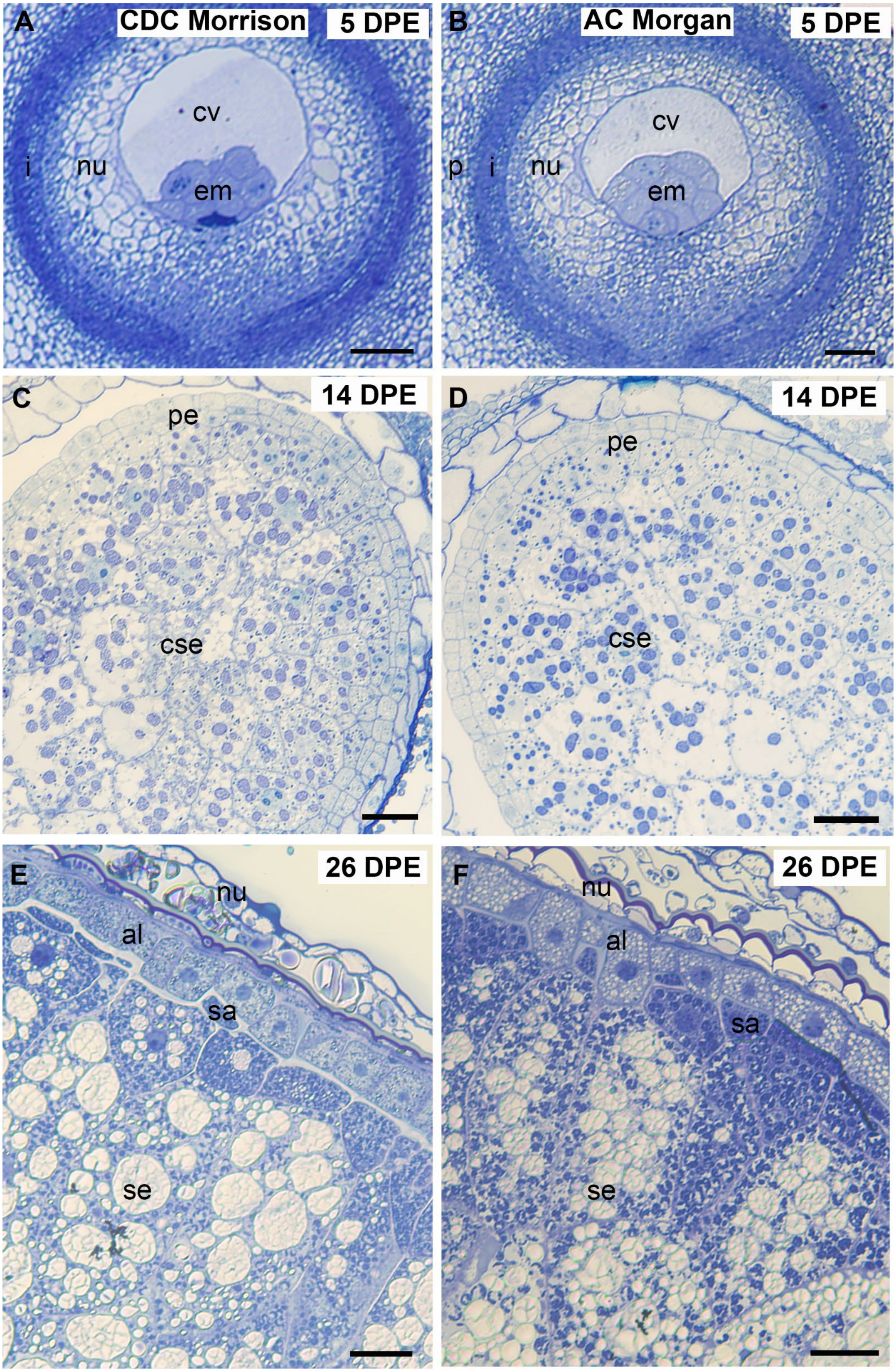

Supplemental Figure S2: Toluidine Blue staining of sections of CDC Morrison and AC Morgan endosperm at 5, 14 and 26 days post-emergence (DPE) for developmental comparison. A. CDC Morrison grain at 5 DPE. The syncytium is round showing a central vacuole and a multicellular embryo surrounded by maternal tissues. **B**. At 5 DPE, a section of AC Morgan grain displays a very similar developmental stage to **A** with a central vacuole and multicellular embryo. **C.** A section through a 14 DPE, CDC Morrison grain at early differentiation showing 2 to 3 layers of small, peripheral endosperm cells and to the inside larger, starch filled central endosperm cells. **D.** A section through AC Morgan endosperm at 14 DPE displaying an almost identical stage of development to **C**. **E.** A section through a 26 DPE CDC Morrison grain showing fully differentiated aleurone and sub-aleurone layers and starchy endosperm cells. **F.** 26 DPE section of AC Morgan endosperm at the end of the differentiation phase as in **E.** al, aleurone; cse, central starchy endosperm; cv, central vacuole; em, embryo; i, integuments; nu, nucellus; pe, peripheral endosperm; sa, sub-aleurone; se, starchy endosperm. Scale bars = 50 µm.

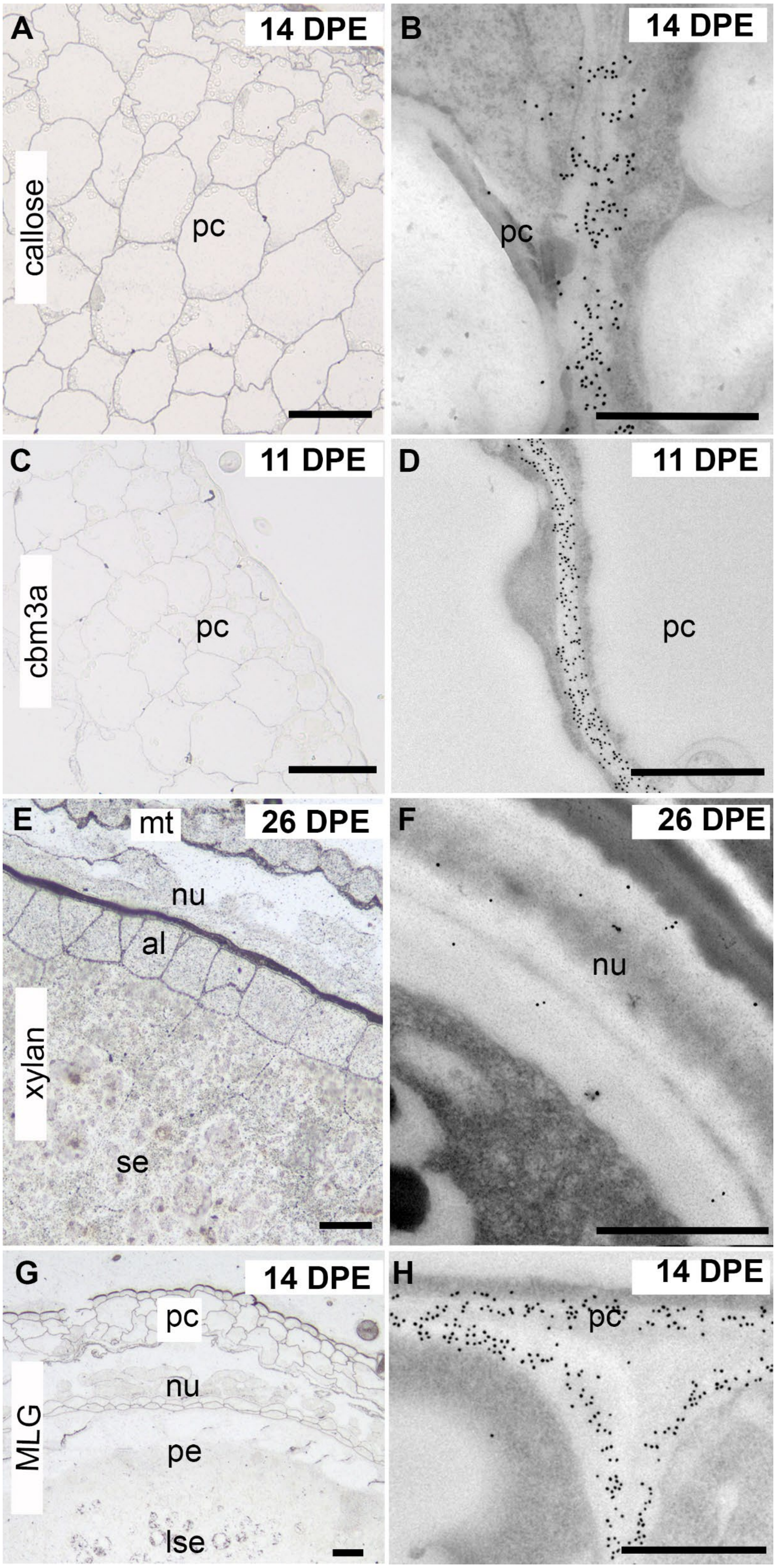

Supplemental Figure S3 . Positive antibody controls showing immunolabelled maternal tissues. A. At 14 DPE, Silver-Enhanced Light Microscopy (SELM) reveals callose along the cell walls of maternal tissues. However, closer examination with **(B)** TEM reveals that callose is restricted to those areas of wall containing plasmodesmata. **C, D**. At 11 DPE, SELM and TEM show that CBM3a labels the integuments strongly, presumably because it binds to MLG in the maternal walls. **E.** The LM11 antibody labels the maternal tissues and the aleurone walls. **F**. TEM image of LM11 labelling in the nucellar cell walls. **G.** At 14 DPE the MLG antibody labels the maternal tissues but no labelling is present in the endosperm cell walls. **H.** TEM image of pericarp cell walls. al, aleurone; lse, starchy endosperm cells located within the lobes; nu, nucellus; pe, peripheral endosperm; mt, maternal tissues; pc, pericarp; se, central starchy endosperm. Scale bar = 50 µm (A, C, E, G), 1 µm (B, D, F, H).

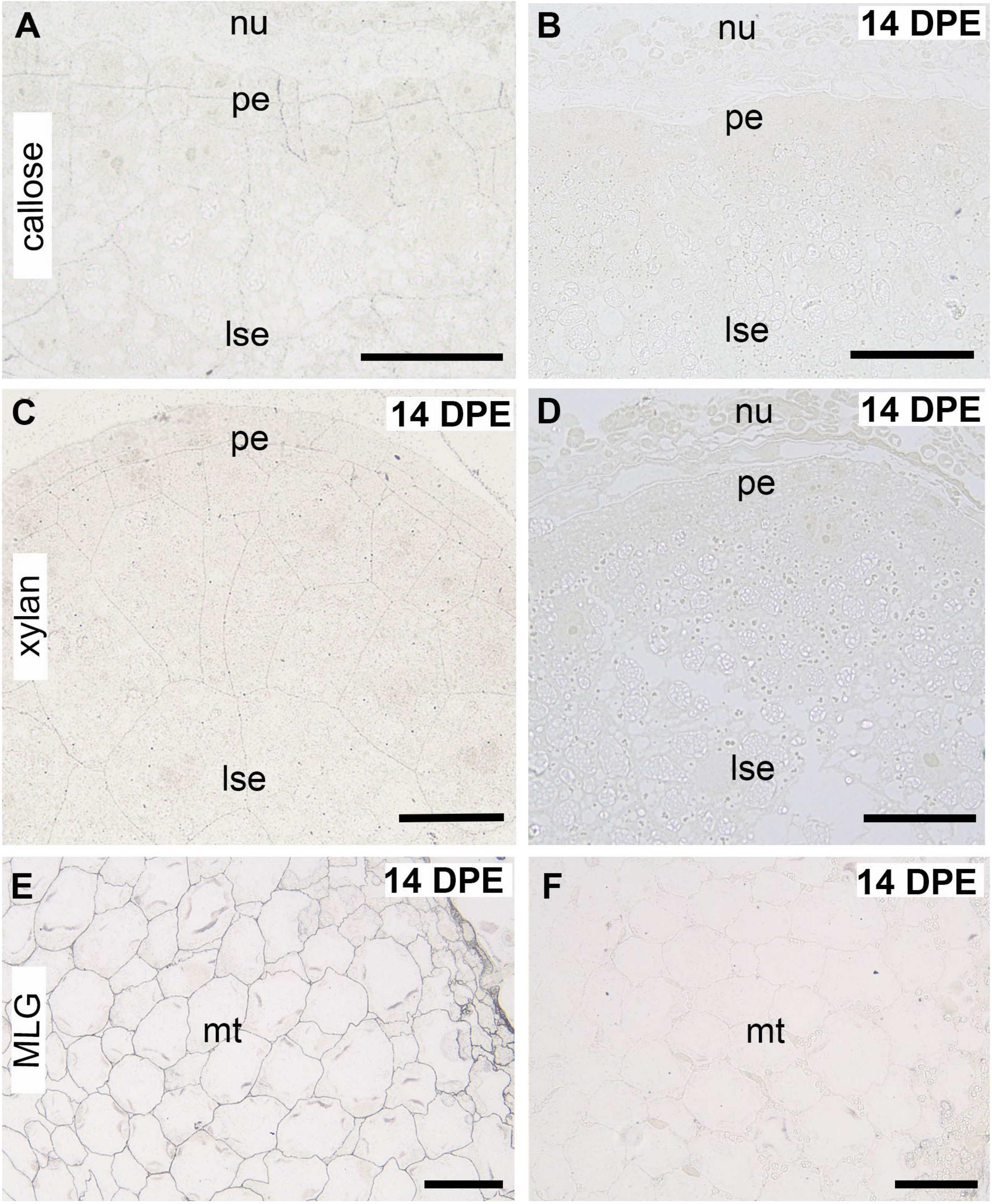

Supplemental Figure S4: Pre-absorption controls. A. Punctate pattern of callose labelling in 14 days post-emergence (DPE) oat endosperm. **B**. No callose labelling is present when the callose antibody preincubated with laminarin is applied to sections. **C.** Xylan labelling after pre-treatment with arabinofuranosidase in 14 DPE oat sections**. D.** No xylan labelling is present when the LM11 antibody pre-incubated with wheat arabinoxylan is applied to sections. **E.** At 14 DPE, MLG is present in the maternal tissues but is absent when the MLG antibody pre-incubated in barley flour is applied to sections (**F**). lse, starchy endosperm cells located within the lobes; mt, maternal tissues; nu, nucellus; pe, peripheral endosperm. Scale bars = 50 µm.

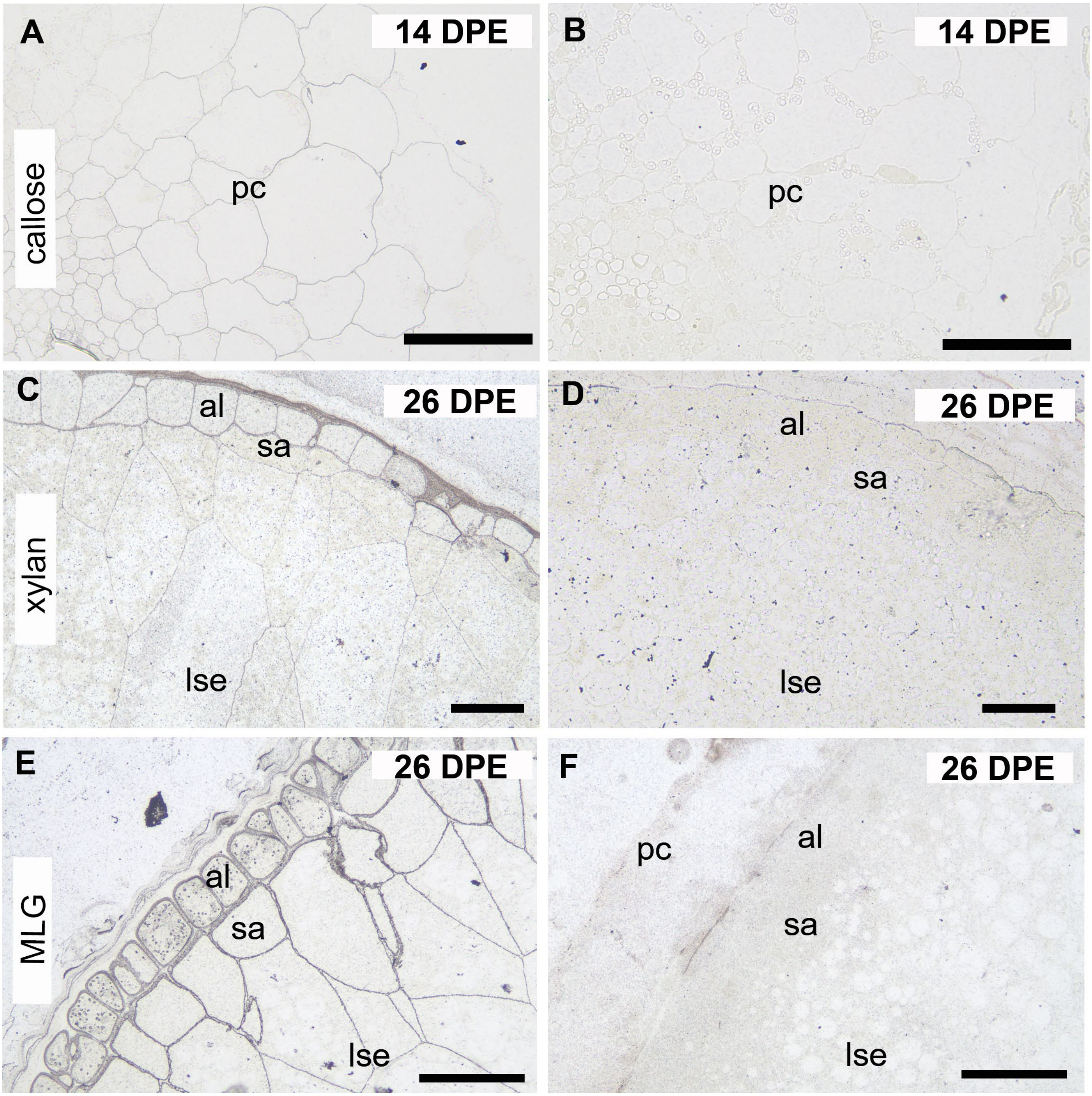

Supplemental Figure S5: Enzyme controls. **A.** Callose labelling in the pericarp of 14 days post-emergence (DPE) grain. **B.** No callose labelling is present when sections are pre-treated with endo-β-1,3-glucanase. **C.** Heteroxylan labelling is strong in 26 DPE endosperm after treatment with arabinofuranosidase. **D.** No heteroxylan labelling is present when sections are pre-treated with arabinofuranosidase followed by xylanase. **E.** Mixed-linkage glucan (MLG) labelling is strong across all cell types of the 26 DPE endosperm**. F.** No labelling is present when sections are incubated in lichenase prior to MLG antibody labelling. al, aleurone; lse, starchy endosperm cells located within the lobes; p, pericarp; sa, sub-aleurone. Scale bar = 50 µm (A, B, C, D), 100 µm (E, F).

Supplemental Table S1: Agronomic and grain quality characteristics of Canadian oat spring varieties CDC Morrison and AC Morgan. Values are data averages calculated from 16 Canadian and U.S.A. oat variety trial sites utilised by PepsiCo between 2017-2020.

**Traits**	**CDC Morrison**	**AC Morgan**
Average yield (kg/ha) *	4341.8 ± 875.6	5178.0 ± 1255.0
Average days to maturity	86.7 ± 9.9	91.5 ± 9.1
Average days to heading	53.4 ± 3.2	54.7 ± 4.0
Average plant height at maturity (cm)	82.0 ± 12.0	92.2 ± 13.2
Average groat (%)	69.6 ± 2.9	69.0 ± 3.1
Average thins (%)	4.4 ± 2.5	2.5 ± 1.5
1000 Kernel weight (g) ^	35.6 ± 0.5	41.8 ± 0.6
Average MLG (%)	5.89 ± 0.43	4.35 ± 0.35
Average protein (%)	17.63 ± 2.48	14.65 ± 1.83
Average fat (%)	7.11 ± 0.70	7.06 ± 0.59

* values derived from small plots within variety trials, adjusted to 13.5% moisture

^ values derived from grain harvested in 2020 from a field site in Nipawin, Saskatchewan, Canada and used in this study

Supplemental Table S2: Monosaccharide linkage data of 'popped' endosperm cell walls of mature grain of CDC Morrison and AC Morgan.

Derivative Linkage	CDC Morrison (mol %)	AC Morgan
t-Glc (p)	1.34	1.19
1,3-Glc (p)	23.18	22.74
1,4-Glc (p)	55.80	55.60
1,6-Glc (p)	0.40	0.68
1,4,6-Glc (p)	0.06	0.15
1,2,6-Glc (p)	0.21	0.26
**Total Glc**	**80.99**	**80.63**
t-Xyl (p)	0.69	0.92
1,4-Xyl (p)	7.07	6.72
1,3,4-Xyl (p)	0.48	0.59
1,2,4-Xyl (p)	0.35	0.20
1,2,3,4-Xyl (p)	1.57	1.48
**Total Xyl**	**10.16**	**9.91**
t-Ara (f)	4.69	4.45
1,5-Ara (f)	0.61	0.79
**Total Ara**	**5.30**	**5.23**
t-Gal (p)	0.20	0.54
1,3-Gal (p)	0.35	0.38
1,6-Gal (p)	0.12	0.03
1,3,4-Gal (p)	0.29	0.08
1,3,6-Gal (p)	0.86	1.17
**Total Gal**	**1.81**	**2.19**
1,4-Man (p)	1.49	1.62
1,4,6-Man (p)	0.14	0.30
1,3,6-Man (p)	0.02	0.06
**Total Man**	**1.65**	**1.98**
t-GlcA (p)	0.09	0.05
**Total GlcA**	**0.09**	**0.05**

Supplemental Table S3: Calculated polysaccharide composition of 'popped' endosperm cell walls of mature CDC Morrison and AC Morgan grain. Calculations are based on the method outlined in Pettolino et al. (2012).

Polysaccharide		Derivative Linkage	CDC Morrison (mol %)	AC Morgan
**Pectin**				
	**Arabinan**	1,5-Ara (f)	0.61	0.79
		**Total Arabinan**	**0.61**	**0.79**
	**Type I AG**	1,4-Gal (p)	0.00	0.00
		1,3,4-Gal (p)	0.29	0.08
		t-Ara	0.29	0.08
		**Total Type I AG**	**0.58**	**0.15**
	**Type II AG**	1,3-Gal (p)	0.35	0.38
		1,6-Gal (p)	0.12	0.03
		1,3,6-Gal (p)	0.86	1.17
		t-Ara	0.51	0.63
		t-Gal	0.20	0.54
		**Total Type II AG**	**2.04**	**2.75**
**(1,3) (1,4)-β-Glucan**		1,3-Glc (p)	21.43	20.46
		1,4-Glc (p)	49.94	47.68
		**Total (1,3) (1,4)-β-Glucan**	**71.37**	**68.14**
**Glucuronoarabinoxylan**		1,4-Xyl (p)	7.07	6.72
		1,2,4-Xyl (p)	0.35	0.20
		1,3,4-Xyl (p)	0.48	0.59
		1,2,3,4-Xyl (p)	1.57	1.48
		t-GlcA	0.09	0.05
		t-Ara	3.89	3.69
		**Glucuronoarabinoxylan**	**13.45**	**12.73**
**Glucomannan**		1,4-Man (p)	1.49	1.62
		1,4-Glc (p)	0.50	0.54
		**Total Glucomannan**	**1.99**	**2.17**
**Xyloglucan**		1,4,6-Glc (p)	0.06	0.15
		1,4-Glc (p)	0.06	0.15
		t-Xyl	0.06	0.15
		**Total Xyloglucan**	**0.17**	**0.44**
**Cellulose**		1,4-Glc	4.12	4.76
		**Total Cellulose**	**4.12**	**4.76**
**Callose**		1,3-Glc (p)	1.75	2.28
		**Total Callose**	**1.75**	**2.28**
		**Total**	**96.08**	**94.21**

Supplemental Table S4: Cell wall antibody probes, substrates and cell wall degrading enzymes used in this study.

<table>
<tr><th>Target polysaccharide</th><th>Primary antibody</th><th>Antibody dilution</th><th>Antibody source</th><th>Antibody substrate</th><th>Antibody substrate source</th><th>Enzyme used to remove target polysaccharide</th><th>Enzyme source</th></tr>
<tr><td>Callose</td><td>(1,3)-β-D-glucan, 400-2</td><td>1:50</td><td>Biosupplies</td><td>Laminarin (Laminaria digitara)
Curdlan (Alcaligenes faecalis)</td><td>Sigma
Megazyme</td><td>(1,3)-endo-β-D-glucanase (Trichoderma sp.), E-LAMSE</td><td>Neogen</td></tr>
<tr><td>MLG</td><td>(1,3;1,4)-β-D-glucan, 400-3</td><td>1:500</td><td>Biosupplies</td><td>Barley flour</td><td>Neogen</td><td>Lichenase ((1,3; 1,4)-endo-β-D-glucanase) (Bacillus subtilis), E-LICHN</td><td>Neogen</td></tr>
<tr><td rowspan="2">Arabinoxylan</td><td rowspan="2">LM11</td><td>1:10*</td><td>Kerafast</td><td rowspan="2">Wheat flour arabinoxylan</td><td rowspan="2">Biosupplies</td><td rowspan="2">(1,4)-endo-β-xylanase M3 (Trichoderma longibrachiatum) E-XYTR3
α-L-arabinofuranosidase (Aspergillus niger) E-AFASE</td><td rowspan="2">Neogen</td></tr>
<tr><td>1:100*</td><td>Biosupplies</td></tr>
<tr><td>Xyloglucan</td><td>LM15</td><td>1:10</td><td>PlantProbes</td><td>Tamarind xyloglucan</td><td>Neogen</td><td>-</td><td>-</td></tr>
<tr><td>Cellulose</td><td>CBM3a</td><td>1:50</td><td>PlantProbes</td><td>Carboxymethyl cellulose</td><td>Neogen</td><td>-</td><td>-</td></tr>
<tr><td>Mannan</td><td>(1,4)-β-D-mannan</td><td>1:50</td><td>Biosupplies</td><td>Guava galactomannan</td><td>Neogen</td><td>-</td><td>-</td></tr>
<tr><td>Arabinogalactan proteins (AGPs)</td><td>JIM8</td><td>1:10</td><td>CarboSource</td><td>-</td><td>-</td><td>-</td><td>-</td></tr>
<tr><td rowspan="2">Homogalacturonan</td><td>LM19</td><td>1:10</td><td>Kerafast</td><td>-</td><td>-</td><td>-</td><td>-</td></tr>
<tr><td>JIM7</td><td>1:10</td><td>CarboSource</td><td>-</td><td>-</td><td>-</td><td>-</td></tr>
</table>

* LM11 antibody sourced from different suppliers was used at different dilutions

Supplemental Materials & Methods:

Milling of oat grains

Whole oat grains harvested in 2020 from a field site in Nipawin, Saskatchewan, Canada were heated in an oven at 130 °C for 90 min. After cooling, the grains were dehulled by placing small batches onto a piece of coarse sandpaper and rubbing them with a sandpaper-covered wooden block. The outer layers of the grains were removed by sieving through a 1 mm sieve. The dehulled grains were then pearled in batches of ~20 g with a Seedburo 109B/C Barley Pearler set to continuous pearling for 40 sec. During this process, a large proportion of grains were broken and some parts of the outer (brown) layers were retained, mostly within the crease. The abraded grains were then milled extensively with a DeLonghi conical burr coffee grinder (#KG521.M) on the finest setting such that the resulting flour passed through a 500 µm sieve.

Nutritional analyses of oat grain flour

To determine MLG content, oat flour (< 500 µm particle size) was used as input into the Mixed Linkage β-Glucan Assay kit (K-BGLU, AACC Method 32-23.01, AOAC Method 995.16) (Megazyme Int., Bray, Ireland). A modified version of the assay based on the method described by McCleary and Codd (1991) was used to analyze isolated cell wall samples (15 mg).

Oat flour starch was quantitated using the Total Starch Assay kit (K-TSTA, AACC Method 76-13.01) (Megazyme), following the manufacturer's instructions. For each sample, assays were performed in duplicate. A small-scale version of the same assay was used to quantitate starch in isolated cell walls (10 mg). Barley and oat flour were included as controls under the same assay conditions.

Total dietary fiber content was determined following the protocol described for the Available Carbohydrates and Dietary Fiber kit (K-ACHDF, AOAC TDF Method 991.43, Megazyme), with some modifications. The amount of starting material was reduced to approximately 250 mg flour (< 500 µm particle size) for each assay with proportionally reduced volumes used thereafter. In addition, the α-amylase digestion step was performed at 80°C not 100°C. For each sample, assays were performed in triplicate.

Protein content of the oat grain flour was determined by the Australian Proteome Analysis Facility (APAF, Macquarie University, NSW, Australia) by liquid hydrolysis in 6 M HCl for 24 h. Each assay was performed in triplicate (1 g of flour fraction per replicate) with technical duplicates.

MLG quantification by HPAEC-PAD analysis

For MLG quantification and DP3:DP4 determination, 10 mg of isolated cell walls was resuspended in 1.6 ml 20 mM sodium phosphate buffer pH 6.5, incubated at 90°C for 30 min, cooled and then digested with 2 U lichenase (E-LICHN, Megazyme) for 1.5 h at 50°C whilst shaking at 1000 rpm in an Eppendorf Thermomixer® C. The reactions were precipitated with four volumes of 100% ethanol overnight at −20°C before centrifugation at 15,000 *g* for 10 min. The resulting supernatant containing the released oligosaccharides was dried under a stream of nitrogen gas and resuspended in 200 µL ultra high quality (UHQ) water. Released oligosaccharides were separated on a CarboPac PA 200 column (Dionex) equilibrated with 25 mM NaOAc in 0.14 M NaOH by high performance anion exchange chromatography with pulsed amperometric detection (HPAEC-PAD) using a Dionex ICS-5000+ (Thermo Fisher Scientific). Oligosaccharides were eluted at 0.45 min/mL with a linear gradient of NaOAc from 25 mM to 275 mM in 0.14 M NaOH over 16 min and quantified from peak area (nC min) using Chromeleon™ 7.2.6 Chromatography Data System software (Thermo Fisher Scientific), with lichenase-digested barley flour supplied in the Mixed linkage β-Glucan Assay kit (K-BGLU, Megazyme) used as a standard under the same chromatographic conditions.

Collection of oat endosperm

Endosperm from oat grains was collected based on the method described by Gartaula et al. (2017) with some minor modifications. Briefly, oat grains were heated in an oven at 130 °C for 90 min to inactivate enzymes. After cooling, grains were soaked in excess water in a beaker (200 mL/100 g grains) and kept in a refrigerator (4 °C) for 5 d and 16 h (CDC Morrison) / 7 d (AC Morgan), with the water changed daily. To collect endosperm tissue, the distal (beard) end of each grain was carefully cut with scissors. The proximal (germ) end was then pressed so that the white endosperm 'popped' out from the grain. Popped endosperm tissue was collected in screw-capped sputum cups and kept on ice.

Preparation of cell walls from popped endosperm

Cell wall isolation

To obtain pure endosperm cell walls, popped endosperm tissue was thoroughly ground in a mortar and pestle and then transferred to a beaker. A minimum amount of 70% v/v ethanol containing 1% w/v SDS was added and then stirred using a magnetic stirrer on a hot plate (37 °C, 500 rpm) for 60 min. The mixture was then subject to wet sieving through a 50-micron nylon mesh to remove any undisrupted tissue, as well as to remove as many starch granules as possible before gelatinisation and enzyme digestion.

Starch gelatinisation & enzyme digestion

The endosperm slurry was centrifuged at 3200 *g* for 10 min and supernatant discarded. The pellet was washed 3 times with cold (4 °C) 1x PBS buffer (pH 7.2) and centrifuged (4 °C, 3200 *g*, 10 min) between each wash. A minimal amount of PBS buffer (pH 7.2) was added to the residue following the washes and mixed thoroughly by hand to ensure there were no clumps. The slurry was then heated to 100 °C on a hot plate for 30 min to gelatinise the starch and denature proteins. Thermostable α-amylase (Megazyme, EBLAAM, 50 µl per gram residue) was added, followed by incubation at 70 °C (with magnetic stirring at 200 rpm) for 4 h. The slurry was equilibrated to 60 °C, then protease (P4860, Sigma-Aldrich, 60 µl per gram) and amyloglucosidase (Megazyme, E-AMGDF, 100 µl per gram) added, followed by further incubation at 60 °C (with magnetic stirring at 200 rpm) for 4 h to hydrolyse proteins and residual starch oligosaccharides, respectively. Before proceeding, starch content was examined by staining a droplet of the slurry with iodine/KI stain under an Olympus light microscope (Tokyo, Japan). If starch was detected, as identified by blue coloration, the enzyme digestion was repeated until no starch was evident.

Alcohol precipitation

The slurry was cooled, concentrated in a rotary evaporator to a small volume and then passed through a 20-micron nylon mesh under 80% v/v ethanol to remove smaller particulate matter. Four volumes of 100% ethanol was added, and the mixture stored overnight at -20 °C. Following centrifugation at 3200 *g* for 5 min at 4 °C, the supernatant was discarded, and the pellet washed twice with cold 100% ethanol. As a final step, the cell walls were air-dried in a vacuum oven with silica beads at room temperature prior to further analysis.

Callose analysis

Callose content was estimated using a modified method based on the Mixed Linkage β-Glucan Assay Kit (K-BGLU, AACC Method 32-23.01, AOAC Method 995.16). One milligram of isolated cell walls was resuspended in 200 mM sodium acetate buffer (pH 5) and boiled for 10 min, followed by sonication for 20 min. Enzymatic digestion with endo-(1,3)-β-glucanase (E-LAMHV, Megazyme) was conducted in an Eppendorf Thermomixer® C (40 °C, 2000 rpm for 24 h). Digestions were stopped by heating samples to 90 °C for 15 min. Laminarin and curdlan (Sigma-Aldrich) were included as controls under the same conditions.

Cellulose analysis (Acetic/Nitric acid assay)

A small-scale estimation of the amount of crystalline cellulose in endosperm cell walls was conducted in duplicate using the method of Updegraff (1969) but determined by weight (100 mg samples) instead of a colorimetric assay.

Monosaccharide linkage analysis

The polysaccharide composition of 'popped' oat endosperm cell walls was performed following the protocol described by Pettolino et al. (2012). Briefly, 10 mg of duplicate samples were subjected to carboxyl reduction to distinguish neutral and acidic sugars followed by two rounds of methylation prior to trifluoracetic acid (TFA) hydrolysis and NaBD4 reduction, and finally acetylation to generate partially methylated alditol acetates (PMAAs). These were then separated and quantified by GC-MS (Agilent, 7890B-5973) using a high polarity BPX70 column. Polysaccharide composition was deduced as described by Pettolino et al. (2012) with 1,3-Glc*p* first assigned to callose according to the (1,3)-β-glucan assay results (see Callose analysis) and the remainder to MLG. Similarly, 1,4-Glc*p* was first assigned to cellulose and starch based on the outputs of acetic/nitric acid and starch assays, respectively.

Bibliography

Gartaula G, Dhital S, Pleming D, Gidley MJ (2017) Isolation of wheat endosperm cell walls: Effects of non-endosperm flour components on structural analyses. Journal of Cereal Science **74:** 165-173

McCleary BV, Codd R (1991) Measurement of (1 → 3),(1 → 4)-β-D-glucan in barley and oats: A streamlined enzymic procedure. Journal of the Science of Food and Agriculture **55:** 303-312

Pettolino FA, Walsh C, Fincher GB, Bacic A (2012) Determining the polysaccharide composition of plant cell walls. Nature Protocols **7:** 1590-1607

Updegraff DM (1969) Semimicro determination of cellulose in biological materials. Analytical Biochemistry **32:** 420-424
